# Renal denervation reduces atrial remodeling in hypertensive rats with metabolic syndrome

**DOI:** 10.1007/s00395-022-00943-6

**Published:** 2022-07-14

**Authors:** Simina-Ramona Selejan, Dominik Linz, Muriel Mauz, Mathias Hohl, Anh Khoa Dennis Huynh, Thimoteus Speer, Jan Wintrich, Andrey Kazakov, Christian Werner, Felix Mahfoud, Michael Böhm

**Affiliations:** 1 Klinik für Innere Medizin III (Kardiologie, Angiologie und Internistische Intensivmedizin), Universitätsklinikum des Saarlandes und Medizinische Fakultät der Universität des Saarlandes, Kirrbergerstr. 100, Geb. 41.1 (IMED), 66421 Homburg/Saar, Germany; 2 Klinik für Innere Medizin IV (Nephrologie und Hochdruckkrankheiten), Universitätsklinikum des Saarlandes und Medizinische Fakultät der Universität des Saarlandes, Homburg/Saar, Germany

**Keywords:** Renal denervation, Metabolic syndrome, Atrial remodeling, RAGE, CML, HMGB1

## Abstract

Atrial fibrillation (AF) is highly prevalent in hypertensive patients with metabolic syndrome and is related to inflammation and activation of the sympathoadrenergic system. The multi-ligand Receptor-for-Advanced-Glycation-End-products (RAGE) activates inflammation-associated tissue remodeling and is regulated by the sympathetic nervous system. Its counterpart, soluble RAGE (sRAGE), serves as anti-inflammatory decoy receptor with protective properties. We investigated the effect of sympathetic modulation by renal denervation (RDN) on atrial remodeling, RAGE/sRAGE and RAGE ligands in metabolic syndrome. RDN was performed in spontaneously hypertensive obese rats (SHRob) with metabolic syndrome compared with lean spontaneously hypertensive rats (SHR) and with normotensive non-obese control rats. Blood pressure and heart rate were measured by telemetry. The animals were killed 12 weeks after RDN. Left atrial (LA) and right atrial (RA) remodeling was assessed by histological analysis and collagen types. Sympathetic innervation was measured by tyrosine hydroxylase staining of atrial nerve fibers, RAGE/sRAGE, RAGE ligands, cytokine expressions and inflammatory infiltrates were analyzed by Western blot and immunofluorescence staining. LA sympathetic nerve fiber density was higher in SHRob (+44%) versus controls and reduced after RDN (-64% versus SHRob). RAGE was increased (+718%) and sRAGE decreased (− 62%) in SHRob as compared with controls. RDN reduced RAGE expression (− 61% versus SHRob), significantly increased sRAGE levels (+162%) and induced a significant decrease in RAGE ligand levels in SHRob (− 57% CML and − 51% HMGB1) with reduced pro-inflammatory NFkB activation (− 96%), IL-6 production (− 55%) and reduced inflammatory infiltrates. This led to a reduction in atrial fibrosis (− 33%), collagen type I content (− 72%), accompanied by reduced LA myocyte hypertrophy (− 21%). Transfection experiments on H9C2 cardiomyoblasts demonstrated that RAGE is directly involved in fibrosis formation by influencing cellular production of collagen type I. In conclusion, suppression of renal sympathetic nerve activity by RDN prevents atrial remodeling in metabolic syndrome by reducing atrial sympathetic innervation and by modulating RAGE/sRAGE balance and reducing pro-inflammatory and pro-fibrotic RAGE ligands, which provides a potential therapeutic mechanism to reduce the development of AF.

**Supplementary Information:**

The online version contains supplementary material available at 10.1007/s00395-022-00943-6.

## Introduction

The prevalence of atrial fibrillation (AF) in patients with metabolic syndrome is elevated compared to non-affected individuals [37]. Insights into the mechanisms are sparse [44]. However, it is known that metabolic syndrome is associated with increased sympathetic activity, which in turn is associated with a higher risk for AF [6]. Renal Denervation (RDN) can modulate sympathetic activation and is currently under investigation as a device-based hypertension treatment [58].

Receptor-for-Advanced-Glycation-End-products (RAGE) is a multi-ligand receptor responsible for pro-inflammatory and pro-fibrotic responses in several cardiovascular pathologies, diabetes and metabolic syndrome [11, 13]. Proteases [40, 62] cleave it into the soluble RAGE (sRAGE), which antagonizes RAGE effects by neutralizing RAGE ligands and also via blocking RAGE itself [42]. Increased plasma levels of sRAGE are associated with a reduction in AF recurrence after pulmonary vein isolation in diabetic patients [25, 61] and inhibitors of AGE formation have been shown to prevent atrial structural remodeling in diabetic rat models [22, 63]. Appropriately, multiple risk factors found in metabolic syndrome are known to predispose to ventricular remodeling [27, 57]. In previous studies, RDN prevented left ventricular interstitial remodeling in a rat model with metabolic syndrome, hypertension and with coexistent renal insufficiency (Spontaneously Hypertensive Obese rats, SHRob) [29, 45]. The SHRob carries a mutation of the leptin receptor, leading to receptor dysfunction and leptin resistance [50] with increasing obesity, hyperinsulinemia and hyperlipidemia beside hypertension. RDN re-established the left ventricular and serum RAGE/sRAGE balance in these rats [45]. In addition, electrophysiological and structural remodeling of the atria makes such rats with metabolic syndrome prone to AF development [15].

Whether changes in atrial RAGE/sRAGE balances can be attained by RDN and whether this influences atrial structural remodeling processes is unknown. Hence, the present study aimed to characterize the influence of the sympathoadrenergic system and its modulation by RDN on atrial interstitial remodeling and on regulation of the RAGE/sRAGE-system and its ligands in metabolic syndrome.

## Materials and methods

### Reagents

M199 medium and fetal calf serum (FCS) were purchased from Gibco/Invitrogen (Karlsruhe, Germany), Penicillin/streptomycin and ß-adrenergic receptor selective antagonists CGP20712A (C231), ICI118.551 (I127) and isoproterenol (I6504) from Sigma-Aldrich (Deisenhofen, Germany). Primary antibodies against the extracellular domain of human RAGE (ab37647), HMGB1 (ab18256), CML (ab27684) and NFkB (ab28856 and ab131485) were from Abcam (Cambridge, UK), against collagen type I from Southern Biotech (1310-01, Southern Biotech, Birmingham, US), against IL-6 (AF 506) and against TNFα (MAB 510) from R&D Systems/Bio-Techne (Wiesbaden, Germany), against F4/80 (#14-4801-82) and against Ly-6G (#14-5931-82) from eBioscience (San Diego, USA). HRP-conjugated secondary antibodies and all other substances used were from Sigma-Aldrich (Deisenhofen, Germany), unless specified otherwise.

### Animals

Male 10-week-old obese spontaneously hypertensive (SHRob; *n* = 16), lean spontaneously hypertensive rats (SHR; *n* = 8) and normotensive Sprague–Dawley rats (controls (Ctrs); *n* = 9) were purchased from Charles River (Sulzfeld, Germany). At the age of 34 weeks, surgical and chemical renal denervation (RDN) was performed in 8 SHRob as described below. At this age, SHRob had fully established a metabolic syndrome with arterial hypertension, hyperinsulinemia, hyperlipidemia and renal insufficiency. SHRob + RDN (*n* = 8) were compared with 8 sham-operated SHRob (SHRob), normotensive controls (Ctr) and lean SHR. The animals were kept in standard cages for the duration of the study and received a standard Chow diet (standard diet No. 1320; Altromin, Lage, Germany) and tap water ad libitum. The animals were killed 12 weeks after RDN. All experiments were performed in accordance with the National Health Guideline for the Care and Use of Laboratory Animals as well as with the Animal Welfare Guidelines and the German law for the protection of animals. The approval of the responsible regional animal ethics committee was obtained.

### Renal denervation

At the age of 34 weeks, surgical and chemical RDN was performed in 8 SHRob as previously described [29]. Shortly, the rats were anesthetized with 2.5% isoflurane and medial laparotomies with peritoneal incisions were performed to approach both kidneys. All visible nerves were cut in the areas of the renal hili, followed by additional stripping of the adventitia from the renal artery to remove remaining nerve fibers. Finally, the renal arteries were moistened with a 20% phenol/ethanol solution for 10–15 min. Sham operations included only kidney exposition without intervention (performed on Ctrs, SHR and SHRob without RDN). Simultaneously to RDN and the sham operations, telemetric sensors were implanted for systolic, diastolic pressure and heart rate acquisition as described before [29].

### Cardiac functional measurements

Left ventricular hemodynamic measurements were performed as final experiments 12 weeks after RDN was performed. Animals were anesthetized as described below, intubated, and lungs were artificially ventilated. Using a Millar Tip catheter (Millar Instruments Inc, Houston, TX), LV pressures were determined. The Millar Tip catheter was inserted from the right carotid artery and advanced into the LV cavity. Data were digitized at a sampling rate of 1000 Hz and recorded with dedicated software (HEM; Notocord, Croissy, France). LV functional parameters such as end-diastolic pressure, the maximal slope of systolic pressure increment (+ dP/dt), the maximal slope of diastolic pressure decrement (− dP/dt) and time constant of LV-pressure drop (tau) were recorded. Animals were killed after completion of hemodynamic measurements by rapid excision of the hearts under continued deep anesthesia, as described below.

In addition, 1 week before killing, magnetic resonance imaging (MRI) assessment of LV ejection fraction could be performed as described in a previous publication [27] in a total of 13 animals without implanted telemetry devices (*n* = 4 Ctr, *n* = 3 SHR, *n* = 3 SHRob and *n* = 3 SHRobRDN).

### Blood and tissue sampling

After having performed the final left ventricular hemodynamic measurements, the rats were killed at the age of 46 weeks under continued deep general anesthesia with isoflurane, xylazine (Rompun®) and ketamine (Ketavet®). Blood was extracted from the aorta and urine from the urinary bladder and stored at − 80 °C for further analysis. Hearts were quickly excised and atrial tissue was either transferred to 4% paraformaldehyde for histological analysis or was snap-frozen in liquid nitrogen and stored at − 80 °C for further analysis. Norepinephrine was measured in kidney tissue by high-pressure-liquid-chromatography (HPLC) as described before [29].

### Histological analysis

Left atrial (LA) and right atrial (RA) tissue was soaked in buffered 4% paraformaldehyde for 24 h and subsequently embedded in paraffin for further histological analysis. Atrial sections of 3 µm thickness were prepared, deparaffined, rehydrated and stained with hematoxylin and eosin (HE) or Picro-Sirius red for further analysis. The Picro-Sirius-Red staining was used for visualization of interstitial fibrosis. The percentage of interstitial collagen was given by the image analysis software (Nicon Instruments Software (NIS)-Elements (BR 3.2) as the ratio of the area positively stained with Picro-Sirius red to the total LA or RA area. In addition, Picro-Sirius red stained sections were additionally analyzed by polarized microscopy for distribution of collagen type I (red-yellow birefringence) and collagen type III (green birefringence). Furthermore, LA and RA sections were additionally stained with hematoxylin and eosin. One hundred cells were measured to determine the cardiomyocyte cellular area in the respective atrial sections.

### Immunofluorescence staining

Immunofluorescence staining of RAGE in atrial tissue: 3 μm thick paraffin sections of atrial tissue were treated with 0.05% citric acid anhydride solution for heat-mediated antigen retrieval. Sections were then incubated overnight at 4 °C and incubated the following day for additional 2 h at 37 °C with the primary antibody diluted 1:300 (Abcam; ab37647, rabbit polyclonal to RAGE), followed by incubation with the corresponding secondary antibody (TRITC-conjugated donkey anti-rabbit IgG, 1:50 dilution (Jackson ImmunoResearch) at 37 °C for 90 min. 1×PBS buffer containing 0.1% Tween 20 was used for the necessary washing steps. The slices were then mounted with DAPI mounting medium (#H-1200, Vector Laboratories Inc, Burlingame, USA) to visualize the cell nuclei, and finally analyzed by fluorescence microscopy using a Nikon Eclipse epifluorescence microscope (Nikon, Germany) with appropriate filters.

Immunofluorescence staining of tyrosine hydroxylase (TH) in atrial tissue: the preparatory steps prior to the actual immunofluorescence staining, incubation times and washing steps are the same as already described above. For TH-staining, slices were incubated with the primary antibody rabbit polyclonal anti-tyrosine hydroxylase in 1:100 dilution (Abcam, #ab112), followed by incubation with the secondary antibody in 1:50 dilution (FITC-conjugated anti-rabbit-IgG Dianova, Hamburg, Germany). Image acquisition and analysis were performed using the visualization tool Aperio ImageScope × 64 (Leica Biosystems, Wetzlar, Germany). Areas of 200 µm^2^ were defined within which interstitial TH-positive axons with DAPI-positive nuclei were counted.

For immunofluorescence staining of F4/80 and LyG6 in LA and RA tissue, slices were either incubated with anti-mouse F4/80 as primary antibody in 1:50 dilution (#14-4801-82, eBioscience) or with anti-mouse Ly-6G in 1:50 dilution (#14-5931-82, Gr-1; eBioscience). Slides were then washed twice with 1×PBS for 5 min and incubated with the secondary antibody conjugated to biotin (Santa Cruz, sc-2041, diluted 1:200) for 30 min. Afterwards, the slides were incubated with labeled streptavidin-HRP antibody diluted 1:100 (#FP1047, Perkin Elmer) and biotinyl tyramide diluted 1:50 (#FP1019, Perkin Elmer). Similar to the TH-staining analysis described above, F4/80-positive or LyG6-positive immune cells with DAPI-positive nuclei were counted for the whole tissue section displayed on the slide and normalized to the total of analysed tissue area.

All histological analyses were performed in a blinded manner with regard to the respective study group identity.

### Immunoblotting

Left atrial and right atrial samples were homogenized with buffer (in mmol/l: Tris 5, EDTA 1, MgCl2 5, pH 8.0, PMSF 1, Leupeptin 1; Aprotinin 5 µg/ml) and mixed 2:1 v/v with SDS-PAGE loading buffer. After denaturation (95 °C, 5 min; except for collagen type I and collagen type III Western blots, where samples were not denaturated), the samples were separated on 10–12% SDS polyacrylamide electrophoresis gels (25 µg tissue/lane) and transferred to nitrocellulose membranes (Protran®, Schleicher and Schuell GmbH, Dassel, Germany) by semi-dry electrophoretic blotting (0.8 mA/cm2). After blocking with 0.1% Western Blocking Reagent (Roche, Mannheim, Germany) membranes were incubated with primary antibodies for RAGE/sRAGE (ab37647, rabbit polyclonal to RAGE, 1:1000 dilution, Abcam, Cambridge, UK), HMGB1 (ab18256, rabbit polyclonal to HMGB1, 1:1000, Abcam), CML (ab 27684, rabbit polyclonal to CML, 1:2000, Abcam), collagen type I (1310-01, goat anti collagen type I, 1:1000, Southern Biotech), collagen type III (1330-01, goat anti collagen type III, 1:1000, Southern Biotech), NFkBp65 phospho (ab28856, rabbit polyclonal to NFkBp65 (phospho S536) 1:1000, Abcam) and total NFkBp65 (ab131485, rabbit polyclonal to NFkB p65, 1:1000, Abcam), IL-6 (AF506, goat polyclonal to rat IL-6, R&D Systems/Bio-Techne) and TNFα (MAB510, mouse monoclonal to TNFα, R&D Systems/Bio-Techne) at 4 °C for 12–16 h. The respective secondary antibodies were incubated for 60 min at room temperature and used at a dilution of 1:10,000. Proteins were visualized by enhanced chemiluminescence (Amersham Pharmacia Biotech, Freiburg, Germany). Membranes were stripped afterwards for GAPDH analysis as loading control: twice stripped for 15 min at 56 °C with stripping buffer (62.5 nM Tris–HCl (pH 6.8), 2%SDS, 0.1 M 2-mercaptoethanol), followed by repeated wash steps with PBS (in mmol/L: NaCl 170, KCl 33, Na2HPO4 40 and KH2PO4 18, pH 7.2), then blocked again in PBS with 5% nonfat dry milk for 120 min at room temperature. Membranes incubated for phospho-NFkB were stripped for total NFkB. Autoradiographs were quantified by imaging densitometry and analyzed by the “LabWorks 4.6” Software (LabWorks Image Acquisition and Analysis Software, UVP BioImaging Systems, Cambridge, UK). Data are presented as arbitrary units (AU) normalized to GAPDH and a control sample.

### Culture conditions of rat cardiomyofibroblasts (H9C2 cell line)

H9C2-cardiomyoblasts (*Rattus norvegicus*) were purchased from ATCC (ATCC CRL 1446, Wesel, Germany; lot number 3426889) and cultured on uncoated 6-well dishes in 2 ml DMEM medium + 10%FBS in humidified air (5% CO2, 37 °C). After reaching 80% confluency, low FBS medium was added to the cells and stimulation experiments with isoproterenol ± ß-adrenergic receptor antagonists were performed.

Every 24 h, the cells were stimulated with isoproterenol (0.1 umol/l) in the presence or absence of ß-adrenergic receptor antagonists with differing selectivity (ß1-selective CGP 201712A, ß2-selective ICI 118.551) for a total stimulation period of 72 h. The ß-blocker was added 30 min prior to isoproterenol. The cell culture supernatant of the stimulated H9C2 cells was asserved every 24 h and analyzed for sRAGE release. The cells were harvested after 72 h of stimulation and processed for cell fractionation: The cell pellets were resuspended in hypotonic buffer (in mmol/l: Tris 5, EDTA 1, MgCl2 5, pH 8.0, PMSF 1, Leupeptin 1; Aprotinin 5 µg/ml), incubated for 15 min at 4 °C, then subjected to 100,000*g* ultracentrifugation (1 h, 4 °C) to obtain a “cytosolic” and “membranous” fraction (pellet). The membrane fraction was resuspended in hypotonic buffer. Membrane fraction and cell culture medium were analyzed for RAGE/sRAGE content by Western blot analysis. The uniform total protein loading on the gel (50 µg/lane) was controlled by Ponceau Red staining (Dianova, Germany).

For analysis of collagen expression, H9C2 cells were additionally pre-treated with recombinant sRAGE (5 ng/ml; BioVendor, Germany) 1 h prior to isoproterenol stimulation every 24 h for a total stimulation period of 72 h as described above, then harvested as homogenates and analyzed by Western blot analysis. The data are presented as arbitrary units (AU) normalized to GAPDH and a control sample, unless otherwise stated.

### Small interfering RNA (siRNA) transfection

H9C2 cells were cultured on uncoated 6-well dishes in 2 ml DMEM medium + 10% FBS in humidified air (5% CO_2_) at 37 °C. To induce RAGE silencing, H9C2 were given a low FBS hunger medium (0,1% FBS) for 24 h after reaching 80% confluence, followed by RAGE siRNA (sc-106985 Santa Cruz Biotechnology) or negative siRNA(sc-36869, Santa Cruz Biotechnology) transfection (15 nM each). siRNA transfection was performed using Opti-MEM I Reduced Serum Medium (31,985,070; Thermo Fisher Scientific) and Lipofectamine RNAiMAX Transfection Reagent (13,778,150; Thermo Fisher Scientific) according to the manufacturer’s specifications. Silencer Select Negative Control No.1 siRNA (sc-36869, Santa Cruz Biotechnology) was used as negative control. 24 h post-transfection, stimulation experiments with isoproterenol in the presence or absence of ß-adrenergic receptor antagonists with differing selectivity were performed as described above.

### Statistical analysis

Results are presented as mean ± SEM. Significance was estimated with two-way-ANOVA with Tukey’s post hoc test for multiple comparisons. Normal distribution of data was tested by Kolmogorov–Smirnov and Lilliefors test. A *p* < 0.05 was considered significant. GraphPadPrism (version 6.0; GraphPad Software, San Diego California, USA) was used for statistical analysis.

## Results

### Metabolic and hemodynamic parameters

Metabolic and ventricular hemodynamic characterizations of the SHRob and SHR models have been recently published [27, 29]. SHRob showed significantly increased body weight compared with SHR and controls. Telemetry analysis revealed increased systolic blood pressure in SHR and SHRob versus Ctr, while heart rate was lower in SHRob. Fasting serum insulin levels were elevated in SHRob with still normal glycated hemoglobin. Triglycerides and cholesterol were also elevated in SHRob (Table 1).

**Table 1:** Metabolic parameters, left atrial interstitial remodeling, RAGE/sRAGE, RAGE ligands

	Ctr	SHR	SHRob	SHRobRDN	*p* values					
					Ctr vs SHR	Ctr vs SHRob	Ctr vs SHRobRDN	SHR vs SHRob	SHR vs SHRobRDN	SHRob vs SHRobRDN
Body weight [g]	592 ± 11 (*n* = 9)	517 ± 24 (*n* = 8)	699 ± 5 (*n* = 8)	683 ± 9 (*n* = 8)	**0.004**	** < 0.0001**	**0.0004**	** < 0.0001**	** < 0.0001**	0.85
Mean systolic arterial blood pressure [mmHg]	118 ± 10 (*n* = 5)	190 ± 6 (*n* = 5)	220 ± 6 (*n* = 5)	185 ± 10 (*n* = 5)	** < 0.001**	** < 0.001**	** < 0.001**	**0.04**	0.99	**0.01**
Heart rate [bpm]	350 ± 11 (*n* = 5)	325 ± 12 (*n* = 5)	285 ± 3 (*n* = 5)	293 ± 6 (*n* = 5)	0.24	**0.0012**	**0.0033**	**0.036**	0.1	0.92
Creatinine [umol/l]	15.8 ± 1.1 (*n* = 9)	25.2 ± 1.1 (*n* = 8)	29.4 ± 0.7 (*n* = 8)	18.5 ± 2.0 (*n* = 8)	**0.0001**	** < 0.0001**	0.47	0.13	**0.006**	** < 0.0001**
HbA1c [%]	3.6 ± 0.06 (*n* = 9)	3.6 ± 0.08 (*n* = 8)	3.8 ± 0.1 (*n* = 8)	3.8 ± 0.06 (*n* = 8)	0.99	0.26	0.32	0.44	0.49	1.0
Fasting insulin [pg/ml]	925 ± 68 (*n* = 9)	1212 ± 256 (*n* = 8)	10,399 ± 907 (*n* = 8)	8692 ± 347 (*n* = 8)	0.98	** < 0.0001**	** < 0.0001**	** < 0.0001**	**0.0001**	0.1
Cholesterol [mmol/l]	3.9 ± 0.09 (*n* = 9)	3.9 ± 0.12 (*n* = 8)	9.7 ± 0.1 (*n* = 8)	9.1 ± 0.3 (*n* = 8)	1.0	** < 0.0001**	** < 0.0001**	** < 0.0001**	** < 0.0001**	0.1
Triglycerides [mmol/l]	1.8 ± 0.07 (*n* = 9)	1.84 ± 0.09 (*n* = 8)	5.8 ± 0.14 (*n* = 8)	5.54 ± 0.14 (*n* = 8)	0.99	** < 0.0001**	** < 0.0001**	** < 0.0001**	** < 0.0001**	0.48
Renal norepinephrine [pg/ml]	83 ± 3.1 (*n* = 9)	94 ± 2.3 (*n* = 8)	103 ± 4.3 (*n* = 8)	12.9 ± 2.7 (*n* = 8)	0.09	0.0007	** < 0.0001**	0.23	** < 0.0001**	** < 0.0001**
LA RAGE [AU/GAPDH]	0.49 ± 0.08 (*n* = 9)	1.53 ± 0.24 (*n* = 8)	3.94 ± 0.6 (*n* = 8)	1.44 ± 0.2 (*n* = 8)	0.24	** < 0.0001**	0.31	**0.0004**	1.0	**0.0003**
LA sRAGE [AU/GAPDH]	7.7 ± 0.1 (*n* = 9)	5.2 ± 0.1 (*n* = 8)	2.4 ± 0.1 (*n* = 8)	6.2 ± 0.1 (*n* = 8)	0.07	** < 0.0001**	0.43	**0.038**	0.71	**0.003**
LA CML [AU/GAPDH]	1.1 ± 0.1 (*n* = 9)	3.6 ± 0.4 (*n* = 8)	4.4 ± 0.7 (*n* = 8)	1.9 ± 0.3 (*n* = 8)	**0.002**	** < 0.0001**	0.54	0.53	0.05	**0.002**
LA HMGB1 [AU/GAPDH]	1.6 ± 0.2 (*n* = 9)	1.9 ± 0.1 (*n* = 8)	4.9 ± 0.6 (*n* = 8)	1.8 ± 0.2 (*n* = 8)	0.92	** < 0.0001**	0.98	** < 0.0001**	0.99	** < 0.0001**
Collagen type I [AU/GAPDH]	0.97 ± 0.1 (*n* = 9)	1.53 ± 0.2 (*n* = 8)	3.73 ± 0.7 (*n* = 8)	1.04 ± 0.08 (*n* = 8)	0.68	** < 0.0001**	1.0	**0.001**	0.76	** < 0.0001**
Collagen type I/collagen type III ratio	1.02 ± 0.09 (*n* = 7)	1.4 ± 0.13 (*n* = 7)	1.9 ± 0.2 (*n* = 8)	1.03 ± 0.08 (*n* = 7)	0.33	**0.004**	1.0	0.14	0.31	**0.003**
PhosphoNFkB/total NFkB ratio	0.16 ± 0.03 (*n* = 9)	1.28 ± 0.24 (*n* = 8)	3.61 ± 0.56 (*n* = 8)	0.19 ± 0.04 (*n* = 8)	0.07	** < 0.0001**	1.0	** < 0.0001**	0.08	** < 0.0001**
LA IL-6 [AU/GAPDH]	7.51 ± 1.8 (*n* = 9)	18.44 ± 5.1 (*n* = 8)	46.99 ± 7.0 (*n* = 8)	21.23 ± 2.0 (*n* = 8)	0.39	** < 0.0001**	0.19	**0.0015**	0.97	**0.0028**
LA TNFa [AU/GAPDH]	1.24 ± 0.3 (*n* = 9)	0.94 ± 0.3 (*n* = 8)	1.13 ± 0.3 (*n* = 8)	0.88 ± 0.3 (*n* = 8)	0.98	0.99	0.85	0.99	0.97	0.93
LA F4/80 + macrophages per mm^2^	16.4 ± 4.2 (*n* = 7)	25.5 ± 4.5 (*n* = 7)	41.7 ± 1.7 (*n* = 8)	23.2 ± 2.5 (*n* = 7)	0.26	**0.0002**	0.47	**0.012**	0.96	**0.0027**
LA Ly6G + neutrophils per mm^2^	1.4 ± 0.4 (*n* = 7)	2.5 ± 0.9 (*n* = 7)	5.2 ± 0.5 (*n* = 8)	2.5 ± 0.3 (*n* = 7)	0.61	**0.0026**	0.7	**0.025**	0.99	**0.0396**
LA Interstitial Fibrosis [%]	5.92 ± 0.4 (*n* = 7)	10.8 ± 1.7 (*n* = 7)	20.6 ± 1.7 (*n* = 8)	13.7 ± 1.6 (*n* = 7)	0.08	** < 0.0001**	**0.003**	**0.0003**	0.48	**0.01**
LA myocyte cell area [µm^2^]	95.4 ± 1.5 (*n* = 7)	102.8 ± 3 (*n* = 7)	129.6 ± 3.8 (*n* = 8)	100.7 ± 2.4 (*n* = 7)	0.27	** < 0.0001**	0.55	** < 0.0001**	0.95	** < 0.0001**
LA TH + nerve fibers per cardiomyocyte	0.25 ± 0.01 (*n* = 7)	0.29 ± 0.03 (*n* = 7)	0.36 ± 0.02 (*n* = 8)	0.13 ± 0.02 (*n* = 7)	0.71	**0.019**	**0.043**	0.17	**0.0065**	** < 0.0001**
EF [%]	64.5 ± 1.3 (*n* = 4)	50.9 ± 0.94 (*n* = 3)	45.8 ± 1.3 (*n* = 3)	54 ± 0.74 (*n* = 3)	** < 0.0001**	** < 0.0001**	** < 0.0001**	**0.021**	0.22	**0.0004**
LVedP [mmHg]	3.9 ± 0.8 (*n* = 9)	6.7 ± 3 (*n* = 8)	17.5 ± 2.3 (*n* = 8)	6.7 ± 1.8 (*n* = 8)	0.82	**0.0062**	0.85	**0.021**	1.0	**0.034**
+ dP/dt [mmHg/s]	7157 ± 269 (*n* = 9)	7616 ± 299 (*n* = 8)	7965 ± 304 (*n* = 8)	8264 ± 391 (*n* = 8)	0.74	0.3	0.09	0.87	0.49	0.91
-dP/dt [mmHg/s]	6430 ± 294 (*n* = 9)	7056 ± 259 (*n* = 8)	4806 ± 269 (*n* = 8)	5641 ± 210 (*n* = 8)	0.34	**0.001**	0.16	** < 0.0001**	**0.0033**	0.13
Tau [ms]	9.4 ± 0.7 (*n* = 9)	12.3 ± 1.2 (*n* = 8)	16.1 ± 1 (*n* = 8)	12.9 ± 1 (*n* = 8)	0.08	** < 0.0001**	**0.024**	**0.013**	0.94	**0.042**
LV weight [g]	1.05 ± 0.04 (*n* = 9)	1.44 ± 0.04 (*n* = 8)	1.77 ± 0.07 (*n* = 8)	1.46 ± 0.1 (*n* = 8)	**0.0015**	** < 0.0001**	**0.0004**	**0.0137**	0.99	**0.0171**
RV weight [g]	0.23 ± 0.02 (*n* = 9)	0.21 ± 0.01 (*n* = 8)	0.21 ± 0.01 (*n* = 8)	0.21 ± 0.01 (*n* = 8)	0.88	0.88	0.8	1.0	1.0	1.0
Lung weight [g]	1.48 ± 0.08 (*n* = 9)	1.59 ± 0.1 (*n* = 8)	1.49 ± 0.09 (*n* = 8)	1.54 ± 0.2 (*n* = 8)	0.94	1.0	0.99	0.95	0.99	0.99

Data are reported as mean +SEM. Bold values indicate statistically significant (*p*<0.05)

EF: left-ventricular ejection fraction; LVedP: Left-Ventricular end-diastolic Pressure; SHR: Spontaneously Hypertensive Rat; SHRob: Spontaneously Hypertensive Obese Rat; SHRobRDN: Spontaneously Hypertensive Obese Rat with renal denervation; AU: Arbitrary Units; RAGE: Receptor for Advanced Glycation End products; sRAGE: soluble Receptor for Advanced Glycation End products; CML: Carboxy-Methyl-Lysine; HMGB1: High Mobility Group Box1 protein; GAPDH: Glyceraldehyde 3-Phosphate Dehydrogenase; RDN: Renal Denervation; bpm: beats per minute; LV: left ventricle; RV: right ventricle; LA: left atrial; TH: tyrosine hydroxylase; IL-6: interleukin 6; TNFα: tumor necrosis factor α; NFkB: nuclear factor kappa-light-chain-enhancer of activated B-cells; +dP/dt: maximal slope of systolic pressure increment; -dP/dt: maximal slope of diastolic pressure decrement; Tau: time constant of LV pressure drop

The effectiveness of renal denervation was indicated by significantly decreased levels of kidney norepinephrine levels. RDN significantly decreased systolic arterial blood pressure and improved kidney function, evoked a numerical, albeit not statistically significant decrease in serum insulin levels and had no effect on lipid levels or body weight (Table 1).

LV-pressure measurements revealed significantly increased LVedP in SHRob compared to Ctr and SHR (17.5 ± 2.3 mmHg in SHRob versus 3.9 ± 0.8 mmHg in Ctr and 6.7 ± 1.8 mmHg in SHR, *p* < 0.025 for both comparisons). Tau as a parameter of diastolic dysfunction was also significantly higher in the SHRob group versus normotensive Ctr (*p* < 0.0001) and SHR (*p* = 0.013) (Table 1). RDN improved both parameters (Table 1: LVedP 6.7 ± 1.8 in SHRobRDN, *p* = 0.034 versus SHRob; Tau 16.1 ± 1 ms in SHRob versus 12.9 ± 1 ms in SHRobRDN, *p* = 0.042). Maximal positive LV-pressure development (+ dP/dtmax) was unchanged between the groups (Table 1). Maximal pressure decay during diastole (− dP/dtmax) was impaired in SHRob (Table 1, 4806 + 269 mmHg/s in SHRob versus 6430 + 294 mmHg/s in Ctr, *p* < 0.0001), but was partially restored after RDN (5641 + 210 in SHRobRDN). In addition, LV ejection fraction was significantly decreased in SHRob (EF 45.8 ± 1.3%) versus Ctr (64.5 ± 1.3%) and SHR (50.9 ± 0.9%) (*p* < 0.0001 for each comparison) and could be significantly increased after RDN (EF 54 ± 0.7% in SHRobRDN, *p* = 0.0004 versus SHRob).

### Atrial structural and interstitial remodeling

**Fig. 1:**
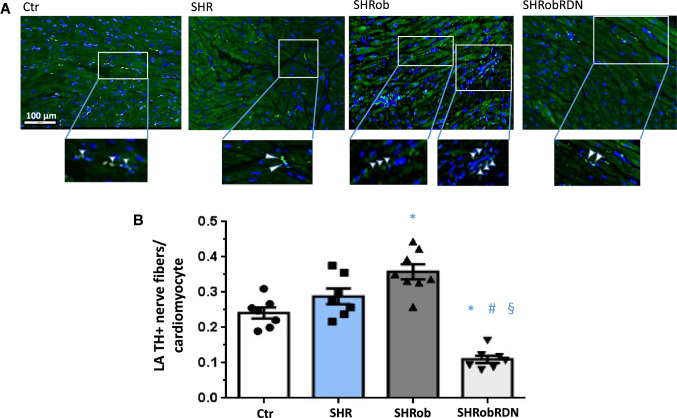
**A** Representative images of immunofluorescence stainings (FITC green; scale bar 100 µm) of left atrial (LA) tyrosinehydroxylase (TH) positive nerve fibers (pointed to by white arrowheads in the enlarged image sections) and **B** quantification of TH + nerve fibers per cardiomyocyte in normotensive controls (*n* = 7), SHR (*n* = 7), SHRob (*n* = 8) and SHRobRDN (*n* = 7). *p < *0.05 versus Ctr; #p* < *0.05 versus SHRob; §p* < *0.05 versus SHR*

Sympathetic innervation of both atria was increased in SHRob as depicted by TH + positive nerve fibers (Table 1 and Supplementary Table 1; Fig. 1 and Supplementary Fig. 1) and could be decreased after RDN (Fig. 1 in LA: 0.36 TH + nerve fibers/cardiomyocyte in SHRob versus 0.13 TH + nerve fibers/cardiomyocyte in SHRobRDN, *p* < 0.0001; Supplementary Fig. 1 in RA: 0.44 TH + nerve fibers/cardiomyocyte in SHRob versus 0.29 TH + nerve fibers/cardiomyocyte in SHRobRDN, *p* = 0.0051).

**Fig. 2:**
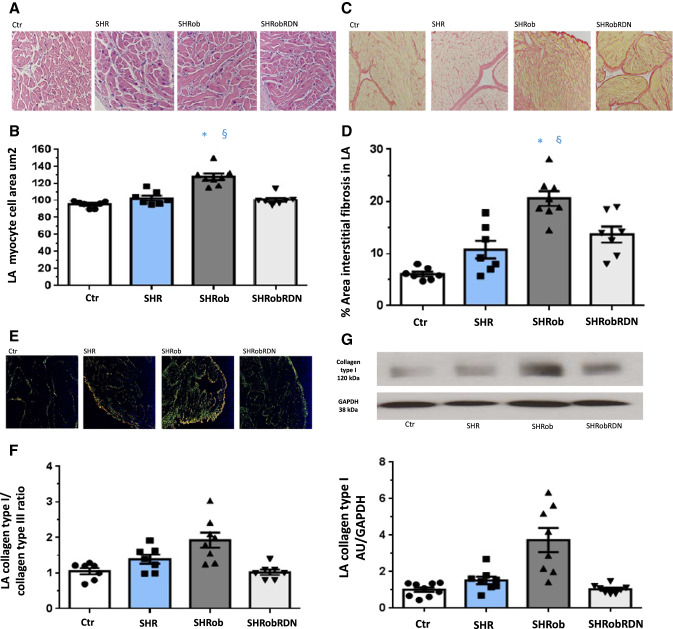
**A** Representative histological pictures (hematoxyline eosin staining; scale bar 50 µm) and **B** Quantification of LA myocyte cell surface in atrial tissue of normotensive controls Ctr (*n* = 7), SHR (*n* = 7), SHRob (*n* = 8) and SHRobRDN (*n* = 7). **C** Representative histological pictures (Picro sirius red staining; scale bar 200 µm) and **D** Quantification of left atrial fibrotic area (interstitial fibrillar collagen fractional area (%)) in normotensive Ctr (*n* = 7), SHR (*n* = 7), SHRob (*n* = 8) and SHRobRDN (*n* = 7). **E** Representative images (polarization microscopy; scale bar 200 µm) and **F** Assessment of collagen type I (red-yellow birefringence)/collagen type III (green birefringence) ratio in left atrial tissue of normotensive Ctr (*n* = 7), SHR (*n* = 7), SHRob (*n* = 8) and SHRobRDN (*n* = 7). **G** Representative Western blot (upper panel) and quantification of collagen type I (lower panel) in left atrial homogenates from normotensive Ctr (*n* = 9), SHR (*n* = 8), SHRob (*n* = 8) and SHRobRDN (*n* = 8). Collagen type I in arbitrary units (AU) normalized to GAPDH. *p < *0.05 versus Ctr; §p* < *0.05 versus SHR; #p* < *0.05 versus SHRob*

In LA SHRob showed increased cardiomyocyte cell area (Table 1; Fig. 2A, B; 129.6 ± 3.8 µm^2^ myocyte cell area in SHRob, *p* < 0.0001 versus Ctr and versus SHR). RDN had anti-hypertrophic effects by almost normalizing atrial myocyte size (100.7 ± 2.4 µm^2^ in SHRobRDN, *p* < 0.0001 versus SHRob and *p* = 0.55 versus Ctr). Interstitial collagen deposition (Table 1; Fig. 2C, D) was only numerically augmented in SHR, but strongly increased in SHRob (20.6 ± 1.7% in SHRob versus 5.9 ± 0.4% in Ctr, *p* < 0.001) and significantly decreased after RDN (13.7 ± 1.6% in SHRobRDN versus 20.6 ± 1.7% in SHRob, *p* = 0.01). In addition, SHRob rats demonstrated a shift to collagen type I (red-yellow fibers) as assessed by polarization microscopy (Table 1; Fig. 2E, F), the collagen type I/collagen type III ratio being normalized after RDN. Western blot analysis for collagen type I protein expression (Table 1; Fig. 2G) confirmed significantly increased collagen type I protein content in SHRob (3.7 ± 0.7 AU/GAPDH in SHRob versus 0.97 ± 0.1 AU/GAPDH in Ctr, *p* < 0.0001; 1.53 ± 0.2 AU/GAPDH in SHR, *p* = 0.0001 versus SHRob), which was reduced after RDN (1.04 ± 0.08 AU/GAPDH in SHRobRDN versus 3.7 ± 0.7 AU/GAPDH in SHRob, *p* < 0.0001). In RA, there were no significant differences with regard to cardiomyocyte cell area (Supplementary Table 1, Supplementary Fig. 2a, b), but there was a comparatively to LA slighter but significantly increased interstitial fibrosis in SHRob with increased collagen I/collagen III ratio as compared with Ctr, both of which could be significantly reduced after RDN (Supplementary Table 1, Supplementary Fig. 2c–f). In addition, similarly to LA, Western blot analysis revealed significantly increased collagen type I expression in SHRob RA (Supplementary Fig. 2 g, h).

### Atrial RAGE/sRAGE regulation

**Fig. 3:**
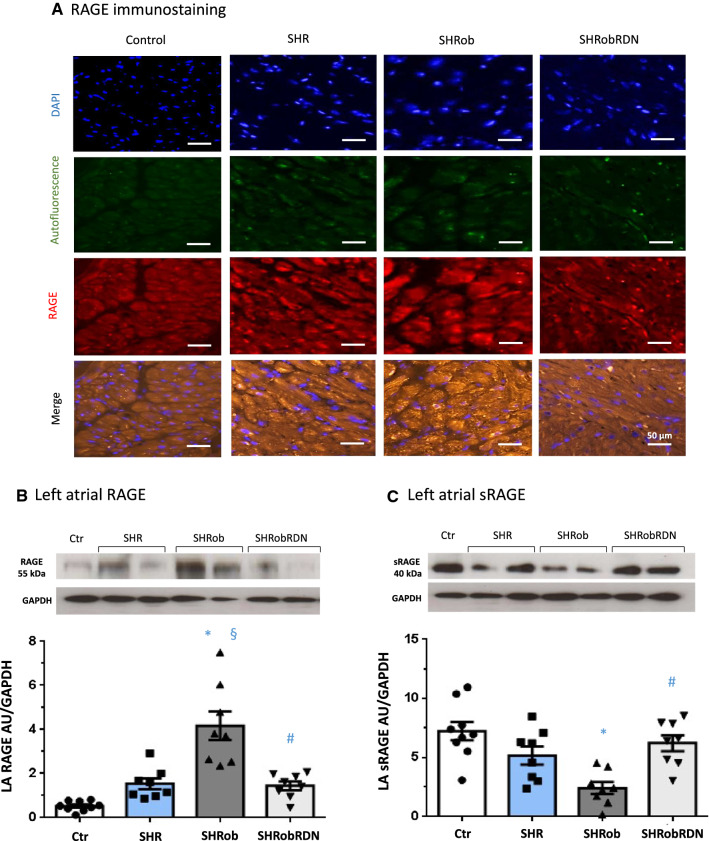
**A** Representative fluorescence microscopy for RAGE (stained red by TRITC), atrial tissue autofluorescence (green) and nuclei (stained blue by DAPI) in left atria of control rats (left panel), SHR (first middle panel), SHRob (second middle panel) and SHRobRDN rats (right panel) (*n* = 3 each group). Magnification 20x. Scale bar 50 µm**. B** Representative Western blot (upper panel) and quantification of left atrial (LA) RAGE (lower panel) and **C** sRAGE in left atrial homogenates from normotensive Ctr (*n* = 9), SHR (*n* = 8), SHRob (*n* = 8) and SHRobRDN (*n* = 8). RAGE and sRAGE in arbitrary units (AU) normalized to GAPDH. *p < *0.05 versus Ctr; §p* < *0.05 versus SHR; #p* < *0.05 versus SHRob*

Assessment of LA RAGE protein expression showed a significant up-regulation in SHRob (Table 1; Fig. 3A, B; 3.9 ± 0.6 AU/GAPDH in SHRob versus 0.49 ± 0.08 in Ctr, *p* < 0.0001). RDN induced a significant reduction in LA content of full-length RAGE in the SHRobRDN group (1.44 ± 0.2 AU/GAPDH, p = 0.0003 versus SHRob). LA sRAGE content was significantly decreased in SHRob as compared to Ctr (Fig. 3C, 2.4 ± 0.1 AU/GAPDH in SHRob versus 7.7 ± 0.1 AU/GAPDH in Ctr, *p* < 0.0001) while in SHRobRDN sRAGE levels were significantly augmented (6.2 ± 0.1 AU/GAPDH, *p* = 0.003 versus SHRob). Analysis of right atrial tissue (RA) rendered similar results with regard to RAGE/sRAGE regulation (Supplementary Fig. 3).

### RDN decreases levels of RAGE ligands CML and HMGB1 in atrial tissue

**Fig. 4:**
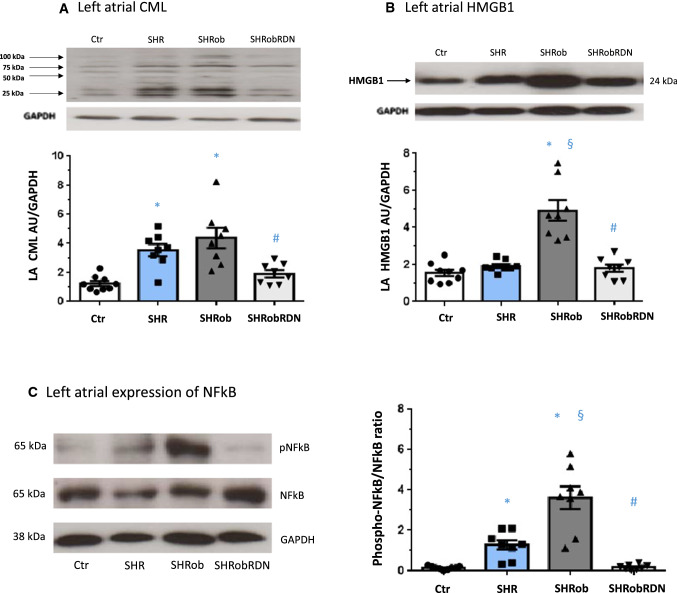
**A** Representative Western blot of left atrial (LA) CML-modified proteins (upper panel) and quantification of left atrial CML in normotensive Ctr (*n* = 9), SHR (*n* = 8), SHRob (*n* = 8) and SHRobRDN (*n* = 8). CML in arbitrary units (AU) normalized to GAPDH. The antibody reacts species independently and specifically recognizes carboxymethyllysine-modified proteins (Western blot with multiple bands belonging to different proteins). **B** Representative Western blot (upper panel) and quantification of left atrial HMGB1 (lower panel) in homogenates from normotensive controls (*n* = 9), SHR (*n* = 8), SHRob (*n* = 8) and SHRobRDN (*n* = 8). HMGB1 in arbitrary units (AU) normalized to GAPDH. **C** Representative Western blots (left panel) and assessment of phospho-NFkB/NFkB ratio (right panel) in left atrial homogenates from normotensive controls Ctr (*n* = 9), SHR (*n* = 8), SHRob (*n* = 8) and SHRobRDN (*n* = 8). *p < *0.05 versus Ctr; § p* < *0.05 versus SHR; #p* < *0.05 versus SHRob*

Left atrial CML levels (Table 1; Fig. 4A) were increased in SHR (3.6 ± 0.4 AU/GAPDH) and SHRob (4.4 ± 0.7 AU/GAPDH) versus Ctr (1.1 ± 0.1 AU/GAPDH in Ctr, *p* < 0.0001 versus SHRob and *p* = 0.002 versus SHR) and attenuated after RDN (1.9 ± 0.3 AU/GAPDH, *p* = 0.002 versus SHRob). Contrary to LA CML regulation, there were no significant differences in right atrial CML levels between the groups (Supplementary Fig. 4a).

SHRob further demonstrated significantly increased left atrial HMGB1 levels as compared with SHR and Ctr (Table 1; Fig. 4B; 4.9 ± 0.6 AU/GAPDH in SHRob versus 1.9 ± 0.1 in SHR and 1.6 ± 0.2 in Ctr, *p* < 0.0001 for each comparison). Again, RDN treatment resulted in a significant reduction of LA HMGB1 protein levels (Fig. 4B; 1.8 ± 0.2 AU/GAPDH in SHRobRDN, *p* < 0.0001 versus SHRob), achieving similarly low levels as in SHR (1.9 ± 0.1 AU/GAPDH) and Ctr alone (1.6 ± 0.2 AU/GAPDH).

The phospho-NFkB/total NFkB ratio, representative of a partially RAGE-induced pro-inflammatory status, was increased in SHRob LA (Table 1; Fig. 4C: 3.6 ± 0.6 in SHRob versus 0.16 ± 0.03 in Ctr, *p* < 0.0001) and was normalized after RDN (0.19 ± 0.04 in SHRobRDN versus 3.6 ± 0.6 in SHRob, *p* < 0.0001, and *p* > 0.99 versus 0.16 ± 0.03 in Ctr).

In the corresponding RA tissue, CML levels were unaltered, but HMGB1 levels showed a similar regulation to LA HMGB1 with significantly increased HMGB1 levels and NFkB activation solely in the SHRob group and with normalization of both parameters after RDN (Supplementary Table 1; Supplementary Fig. 4a–c).

### RDN decreases IL-6 levels and inflammatory infiltrates in atrial tissue

**Fig. 5:**
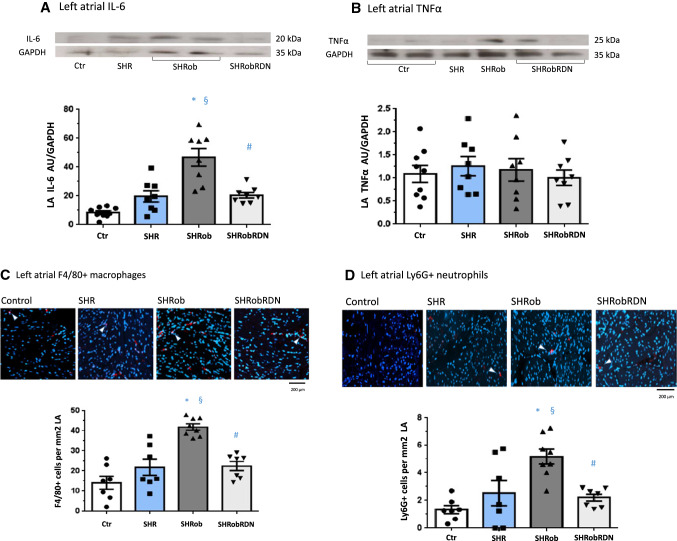
**A** Representative Western blot (upper panel) and quantification of left atrial (LA) IL-6 (lower panel) in homogenates from normotensive Ctr (*n* = 9), SHR (*n* = 8), SHRob (*n* = 8) and SHRobRDN (*n* = 8). IL-6 in arbitrary units (AU) normalized to GAPDH. **B** Representative Western blot (upper panel) and quantification of left atrial TNFα (lower panel) in homogenates from normotensive Ctr (*n* = 9), SHR (*n* = 8), SHRob (*n* = 8) and SHRobRDN (*n* = 8). TNFα in arbitrary units (AU) normalized to GAPDH. *p < *0.05 versus Ctr; § p* < *0.05 versus SHR; #p* < *0.05 versus SHRob.* Representative images of immunofluorescence stainings (TRITC red) for **C** F4/80 macrophages (upper panel; example target cells pointed to by white arrowheads; scale bar 200 µm) and quantification of F4/80 + cells per mm^2^ LA area (lower panel). Nuclei of cells were stained with DAPI (blue). Representative images of immunofluorescence stainings (TRITC red) for **D** Ly6G + neutrophils (upper panel; example target cells pointed to by white arrowheads; scale bar 200 µm) and quantification of Ly6G + cells per mm^2^ LA area (lower panel) in normotensive Ctr (*n* = 7), SHR (*n* = 7), SHRob (*n* = 8) and SHRobRDN (*n* = 7) rats. Nuclei of cells were stained with DAPI (blue). *p < 0.05 versus Ctr; ^*§*^*p* < 0.05 versus SHR; ^*#*^*p* < 0.05 versus SHRob

We observed increased LA IL-6 levels in SHRob as compared with Ctr and SHR (Table 1; Fig. 5A 47 ± 7 AU/GAPDH in SHRob versus 18.44 ± 5.1 in SHR and 7.5 ± 1.8 in Ctr, p < 0.005 for both comparisons). RDN significantly decreased LA IL-6 in SHRobRDN (Fig. 5A, 21.2 ± 2 AU/GAPDH in SHRobRDN, *p* = 0.003 versus SHRob). RA IL-6 levels were likewise significantly increased in SHRob (Supplementary Table 1, *p* = 0.014 versus Ctr), and decreased after RDN (Supplementary Fig. 4d, *p* = 0.009 versus SHRob). SHR showed in both atria only a numerical increase in IL-6 without reaching statistical significance Table 1 and Fig. 5A, Supplementary Table 1 and Fig. 4D). In both atria, TNFα levels were similar between all groups (Fig. 5B and Supplementary Fig. 4e).

Analyzing F4/80-positive macrophages and Ly6G positive neutrophils, we observed a greater myocardial infiltration with those immune cells in both left and right SHRob atria, in both cases with reduced infiltration after RDN (Table 1 and Supplementary Table 1; Fig. 5C and D, Supplementary Fig. 4f, g).

### In vitro ß-adrenergic stimulation of H9C2 cells increases RAGE expression and decreases sRAGE secretion

**Fig. 6:**
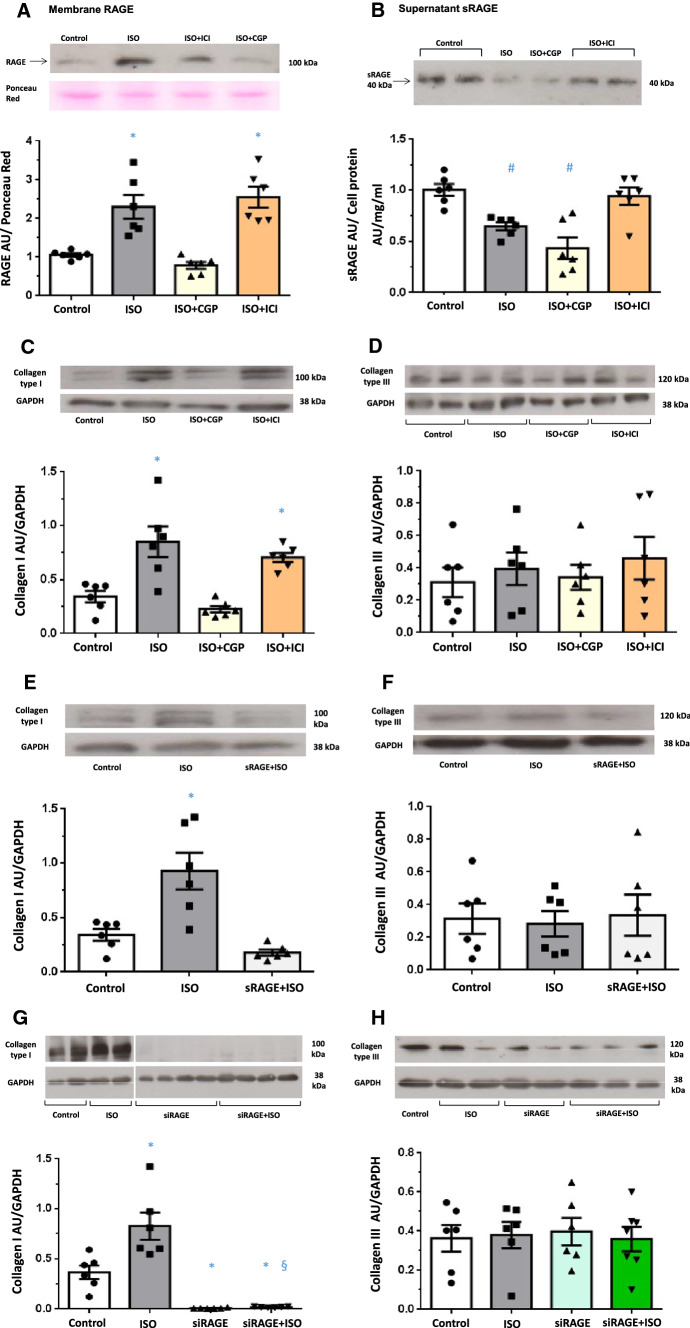
**A** Representative Western blot (upper panel) and quantification of membrane RAGE expression in cardiomyoblasts H9C2 (lower panel) repeatedly stimulated with isoproterenol (ISO) 0.1 umol/l (*n* = 6) in the presence or absence of CGP (ß1-selective antagonist; 0.3 umol/l) or ICI (ß2-selective antagonist; 0.1 umol/l) for 72 h every 24 h. RAGE expression in arbitrary units (AU) normalized to Ponceau Red. **B** Representative Western blot (upper panel) and quantification of sRAGE secretion in cell culture medium of cardiomyoblasts H9C2 (lower panel) repeatedly stimulated with isoproterenol (ISO) 0.1 umol/l (*n* = 6) in the presence or absence of CGP (ß1-selective antagonist; 0.3 umol/l) or ICI (ß2-selective antagonist; 0.1 umol/l) for 72 h every 24 h. sRAGE expression in arbitrary units (AU) normalized to the corresponding cell protein concentration of the cell homogenate. *p < 0.05 versus Control and ISO + CGP; ^*#*^*p* < 0.05 versus Control and ISO + ICI*.* **C** Representative Western blot (upper panel) and quantification of collagen type I expression (lower panel) and **D** collagen type III expression in H9C2 cell homogenates repeatedly stimulated with isoproterenol (ISO) 0.1 umol/l (*n* = 6) in the presence or absence of CGP (ß1-selective antagonist; 0.3 umol/l) or ICI (ß2-selective antagonist; 0.1 umol/l) for 72 h every 24 h. Data are presented as arbitrary units (AU) normalized to GAPDH. *p < 0.05 versus Control and ISO + CGP. **E** Representative Western blot (upper panel) and quantification of collagen type I expression (lower panel) and **F** collagen type III expression in H9C2 cell homogenates repeatedly stimulated with isoproterenol (ISO) 0.1 umol/l (*n* = 6) in the presence or absence of recombinant sRAGE (5 ng/ml). sRAGE was added 1 h before ISO stimulation. Data are presented as arbitrary units (AU) normalized to GAPDH. *p < 0.05 versus Control and sRAGE + ISO*.* **G** Representative Western blot (upper panel; images from different parts of the same gel) and quantification of collagen type I expression (lower panel) and **H** representative Western blot (upper panel) and quantification of collagen type III expression in H9C2 cells transfected with siRNA for RAGE (siRAGE) or control H9C2 cells transfected with scramble RNA and repeatedly stimulated with isoproterenol (ISO) 0.1 umol/l (*n* = 6–7) for 72 h every 24 h. *p < 0.05 versus Control, ^*§*^*p* < 0.05 versus siRAGE

H9C2 cells were repeatedly stimulated with the ß1 + ß2-adrenoreceptor agonist isoproterenol (0.1 µmol/l every 24 h), which lead to a significant increase in RAGE content in the cell membrane (+ 129% (Fig. 6A)). Simultaneously, sRAGE shedding into the cell culture medium was reduced by isoproterenol treatment (− 36% (Fig. 6B)). To identify the ß-adrenergic receptor subtype involved, we also used ß-adrenergic receptor antagonists with different selectivity (CGP 201712A (ß1-selective) or ICI 118.551 (ß2-selective)) simultaneously to isoproterenol stimulation. The isoproterenol-induced RAGE expression (Fig. 6A) was reversed by the ß1-adrenergic receptor blockade with CGP, while sRAGE shedding recovered with ß2-adrenergic receptor blockade by ICI (Fig. 6B).

### Collagen type I expression by H9C2 cells is RAGE dependent

Isoproterenol treatment enhanced collagen type I expression in H9C2 cardiomyocytes (Fig. 6C), but not when preceded by blockade with the ß1-adrenoreceptor-specific blocker CGP, while blockade with the ß2-adrenoreceptor-specific antagonist ICI did not hamper with the isoproterenol-induced effect (Fig. 6C). Contrary to collagen type I, collagen type III expression was unaltered in H9C2 cells exposed to isoproterenol (Fig. 6D).

Pretreatment of H9C2 cells with sRAGE completely inhibited the isoproterenol-induced increase in collagen type I expression (Fig. 6E), with no effect on collagen type III content (Fig. 6F). Likewise, collagen type I production was almost completely abolished in RAGE siRNA transfected H9C2 cells (Fig. 6G), with only a slight but still significant increase in collagen type I content after isoproterenol stimulation of RAGE siRNA transfected cells (Fig. 6G). There was no effect of RAGE silencing on collagen type III expression in H9C2 cells (Fig. 6H).

Thus, both ß1-adrenoreceptor inhibition by CGP and RAGE inhibition by sRAGE or RAGE silencing RNA inhibited the isoproterenol-induced increase in collagen type I production.

## Discussion

This study shows that RDN effectively inhibits atrial sympathetic innervation and atrial remodeling in metabolic syndrome. We observed a significant increase in LA myocyte cell area in SHRob as compared with controls and SHR, which is in agreement with a previous study measuring myocyte diameter [15], while SHR showed only a numerical non-significant increase in myocyte area in our study. In RA, there was no difference in myocyte size between the groups. RDN led to a normalization in left atrial cardiomyocyte size.

Interstitial fibrosis is another characteristic of atria, prone to develop AF [20]. Herein, we documented increased interstitial fibrosis both in the LA and RA of hypertensive rats with metabolic syndrome. RDN effectively inhibited atrial remodeling and reduced atrial fibrosis in these rats. Considering the polarized microscopy and Western blot analyses for collagen type I, the main part of the fibrosis appeared to be caused by an increase in type I collagen with augmented collagen type I/collagen type III ratio. This collagen-shift has been studied in left ventricular stiffness [12], but also in atrial remodeling, where an augmented collagen type I/type III ratio in patients undergoing heart surgery was associated with an increased risk for postoperative atrial fibrillation [14].

RDN has already been shown to decrease at least ventricular interstitial fibrosis and to improve cardiac function in several models of heart failure, whereby several relevant mechanisms of action have been identified [19, 23, 24, 32, 39, 54, 66, 67]. In this regard, it must be emphasized that RAGE itself is known to increase collagen type I transcription [38]. We observed a shift in RAGE/sRAGE balance with increased RAGE and decreased sRAGE in both the left and right atria of sham-operated SHRob, matching the pro-fibrotic changes. RAGE/sRAGE was brought to a more favorable ratio following neuromodulation via RDN. As shown by the in vitro experiments, these effects on RAGE/sRAGE balance are partly independent of systemic blood pressure, but can be induced by sole ß-adrenergic activation. The observations extend previous results [45], where a ß-adrenergic-induced in vitro RAGE/sRAGE regulation in cardiac fibroblasts and peripheral blood mononuclear cells was demonstrated. We could reproduce those results herein with rat cardiomyoblastoma cells (H9C2 cell line). Furthermore, we demonstrate that especially collagen type I expression in H9C2 cells is ß1-adrenoreceptor and RAGE dependent and can be inhibited by sRAGE. RAGE has been described to directly interact with the ß1-adrenoreceptor in cardiomyocytes by forming receptor heterodimers and thus influencing certain signal transduction pathways [68], with the ß1-adrenoreceptor-RAGE heterodimer being blocked by sRAGE. This observation fits with our H9C2 cell culture in vitro results, where we were able to inhibit isoproterenol-induced collagen type I production by H9C2 cardiomyofibroblasts both using specific ß1-AR blockade and sRAGE. Silencing of RAGE in these cells by transfection with RAGE silencing RNA prevented almost any collagen type I expression, while collagen type III expression remained largely unchanged.

In addition, we investigated potential RAGE activators in our rat models and describe increased levels of the RAGE ligand HMGB1 both in the right and in left atria of SHRob, while CML levels were only increased in the left atrium and not in the right atrium, which is supposed to be independent from blood pressure changes. This indicates a certain dependency on blood pressure even in the absence of diabetes with regard to CML. CML-modified proteins seem to accumulate over time not only due to increased serum glucose levels but also due to long-term oxidative stress [9, 16]. It is also known that RAGE activation by RAGE ligands increases oxidative stress and inflammation, which in turn leads to an increased formation of RAGE ligands and transcription of RAGE protein, in the sense of a vicious circle [17]. CML is a major advanced glycation end product and is not only increased in diabetes [43], but also associated with an early development of hypertension [2, 53]. In this study, atrial CML levels were increased both in SHRob and SHR left atria. Similar CML regulations in serum and left ventricular myocardium in this rat model were reported previously [45]. In addition, deposits of AGEs like CML not only promote fibrosis formation by, e.g., RAGE-mediated inflammation and collagen production, but also inhibit collagenase mediated collagen degradation by cross-linking the fibrils, making fibrosis more persistent [3, 65]. This might be one of the reasons for the altogether lower content of fibrosis in SHRob RA when compared with SHRob LA.

HMGB1, the other RAGE ligand and alarmin molecule investigated, is released by activated immune cells or necrotic cells [33]. Recent data suggest that HMGB1 acts as an adipokine in metabolic syndrome [46, 64] and plays a central role in the pathogenesis of insulin resistance [55]. Corresponding to a systemic effect, atrial HMGB1 content was also increased solely in obese animals, as shown in this study, and in left ventricular myocardium and serum according to previous findings [45]. An increase in atrial HMGB1 tissue concentration is of particular interest, as HMGB1 is also associated with pro-thrombotic states [10] and intra-cardiac thrombus formation by activating platelets [59]. Atrial HMGB1 was normalized after RDN in this study, providing evidence for a systemic sympathoadrenergic mediated HMGB1 regulation in metabolic syndrome. Inflammatory mechanisms are known to induce atrial arrhythmias and to promote the development of persistent atrial fibrillation [7]. HMGB1 was increased in both left and right atrial tissue in metabolic syndrome, indicating activation of RAGE with consecutive activation of pro-inflammatory signal transduction pathways. In line with this hypothesis, the NFkB signal transduction pathway was activated in both atria of SHRob and was significantly decreased after RDN. Furthermore, HMGB1 is known to stimulate macrophage migration modulating pro-inflammatory mediators and enhancing the accumulation of immune cells [33]. Analyzing F4/80-positive macrophages and Ly6G positive neutrophils, we detected a greater myocardial infiltration with immune cells in both left and right SHRob atria, in both cases with reduced infiltration after RDN and matching the respective HMGB1 regulation. In addition, we observed an up-regulation of IL-6 in both SHRob atria with improvement after RDN. Similar effects of RDN on inflammatory responses have been recently reported in a mouse model of myocardial ischemia–reperfusion injury [49], which suggests cross-model immunomodulatory effects of RDN.

In summary, atrial RAGE/sRAGE regulation in our SHRob rat model seems to be largely blood pressure independent by showing the same regulation both in the left and right atrium. The same appears to be true for the RAGE ligand HMGB1. Interestingly, NFkB activation follows the same pattern, as does atrial content of the pro-inflammatory cytokine IL-6 and the atrial infiltration with pro-inflammatory immune cells like macrophages and neutrophils.

As mentioned above, RAGE is supposed to play a role in fibrosis formation, considering our in vitro results and additional studies by others [38]. Nevertheless, the amount and differential type and distribution of its ligands as well as the accompanying inflammation play a decisive role in how severe this fibrosis will ultimately be. Fibrosis formation was less pronounced in RA as compared to LA; nevertheless, there was a similar regulation between the groups with significantly increased fibrosis in SHRob both in LA and RA and a significant reduction in fibrosis after RDN. The same applies to the RAGE/sRAGE regulation. The HMGB1 increase was proportionately and relatively greater in LA than in RA, the same holds true for the NFkB activation, inflammatory infiltrates and IL-6 production, so that one can postulate that although some changes such as RAGE/sRAGE regulation may not in principle be blood pressure dependent, hypertension directly or indirectly potentiates the changes, possibly due to the increase in AGEs such as CML in the left atrium, which in turn would potentiate RAGE expression.

The SHRob rat model used herein comprises multiple concomitant risk factors, which might exacerbate atrial structural remodeling including arterial hypertension [26], obesity with hyperinsulinemia [34], and chronic kidney disease [47, 56]. All these conditions are known to be associated with a RAGE/sRAGE imbalance [22, 41], contributing to atrial maladaptive remodeling processes [35, 63]. The association between increased sympathetic nerve activity and a high prevalence of atrial fibrillation has been well demonstrated [28, 30–32]. The activation of the sympathetic nervous system plays an important role in the initiation and perpetuation of atrial fibrillation under various pathophysiological conditions [44] by structural and electrophysiological changes [18, 52, 60]. Albeit we did not measure AF inducibility in this study, telemetry recordings revealed no spontaneous atrial fibrillation. Still, in a previous report using the same rat model for metabolic syndrome, AF inducibility by transesophageal rapid pacing was significantly increased in SHRob as a consequence of electrophysiological and structural remodeling [15].

Catheter-based RDN is currently being utilized for treatment of uncontrolled hypertension [4, 5]. Recently published randomized, sham-controlled clinical trials [1, 21] have provided the proof of concept for the blood pressure-lowering efficacy of this approach. A growing body of evidence suggests, that catheter-based RDN may also be used in patients with cardiac arrhythmias [8, 48, 51]. In a randomized controlled study in 302 patients with paroxysmal atrial fibrillation and hypertension, renal denervation added to pulmonary vein isolation, compared with catheter ablation alone, significantly increased the likelihood of freedom from atrial fibrillation at 12 months [48]. Our findings extend these data by showing that RDN could represent an alternative therapy in atrial fibrillation by inhibiting atrial interstitial remodeling and atrial RAGE/sRAGE dysbalance as well as inflammation in metabolic syndrome.

### Limitations

The animal model for metabolic syndrome includes several individual risk factors and pathologies, i.e. hypertension, disturbed glucose tolerance or renal dysfunction. Each of those could have impacted some of the observations made. From clinical studies, we know that aggressive risk factor management improves long-term success of AF ablation [36]. We aimed to investigate the impact of arterial hypertension alone on the overall picture of the metabolic syndrome regarding atrial RAGE/sRAGE regulation and atrial remodeling by including the SHR group in our study. However, further detailed investigations on obesity, diabetes and renal failure models alone are necessary to characterize the intricate network of pathologies and the influence of the sympathetic nervous system on atrial structural remodeling in each context. Another limitation of this study is that inducibility of atrial fibrillation was not directly measured. Only spontaneous atrial fibrillation was ruled out by telemetry recording analysis.

## Conclusion

Sympathoadrenergic activation in metabolic syndrome worsens RAGE/sRAGE balance leading to interstitial remodeling and damage both in the left and right atrium. Renal denervation improves atrial interstitial remodeling with restored RAGE/sRAGE balance, reduced RAGE ligands and exhibits anti-inflammatory, anti-hypertrophic and anti-fibrotic effects. Our results can help to establish new therapeutic targets and strategies to improve and prevent atrial fibrillation in metabolic syndrome.

## Supplementary Information

Below is the link to the electronic supplementary material.

Supplementary file1 (PDF 4351 KB)

Supplementary file2 (DOCX 15 KB)

Supplementary file3 (DOCX 28 KB)
