# Supplementary material for: Renal denervation reduces atrial remodeling in hypertensive rats with metabolic syndrome
Source: Basic Res Cardiol. 2022 Jul 14;117(1):36. doi: 10.1007/s00395-022-00943-6 (PMC9283368; doi:10.1007/s00395-022-00943-6)

Supplementary Figure 1

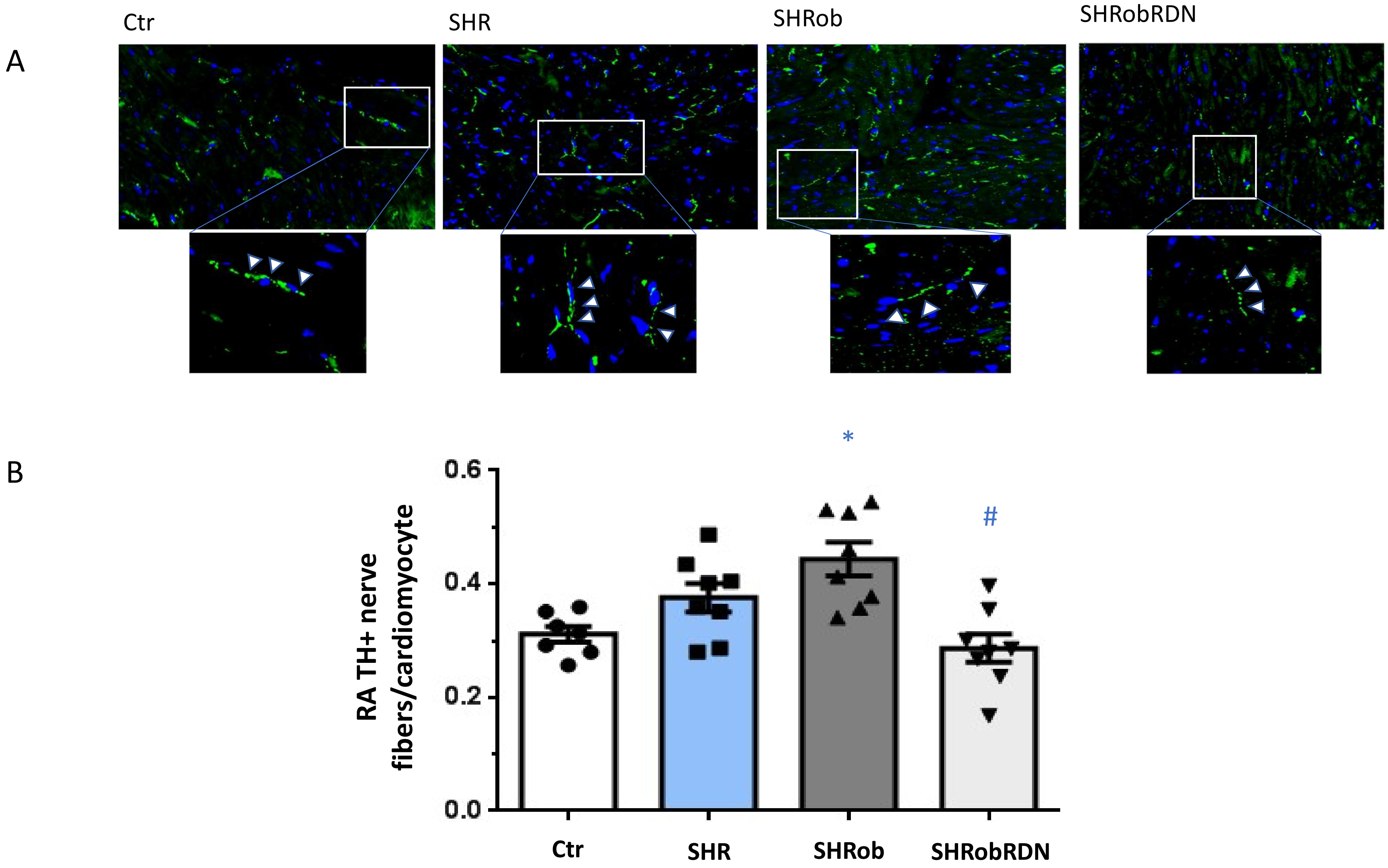

Supplementary Figure 2

A Right atrial myocyte hypertrophy

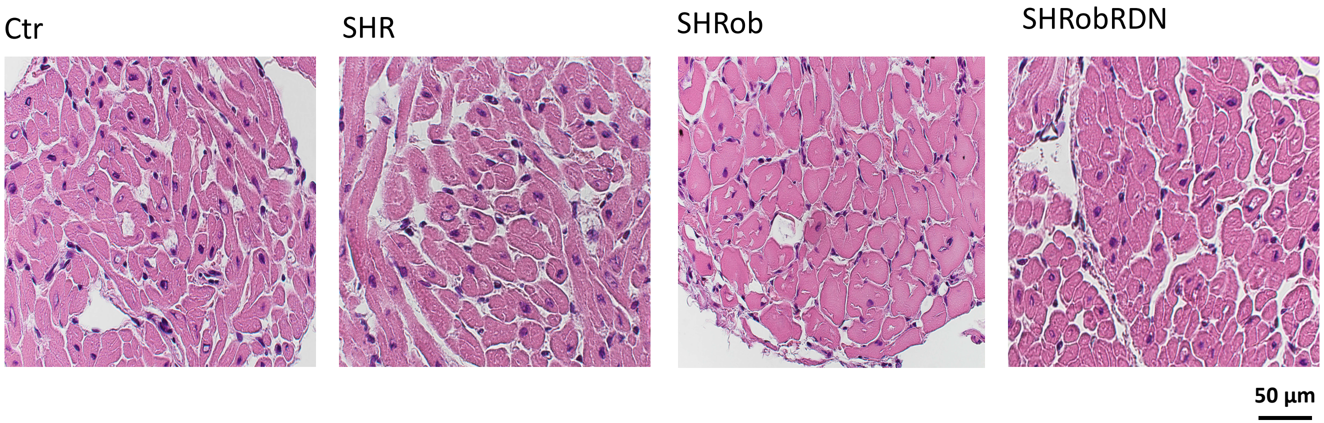

B

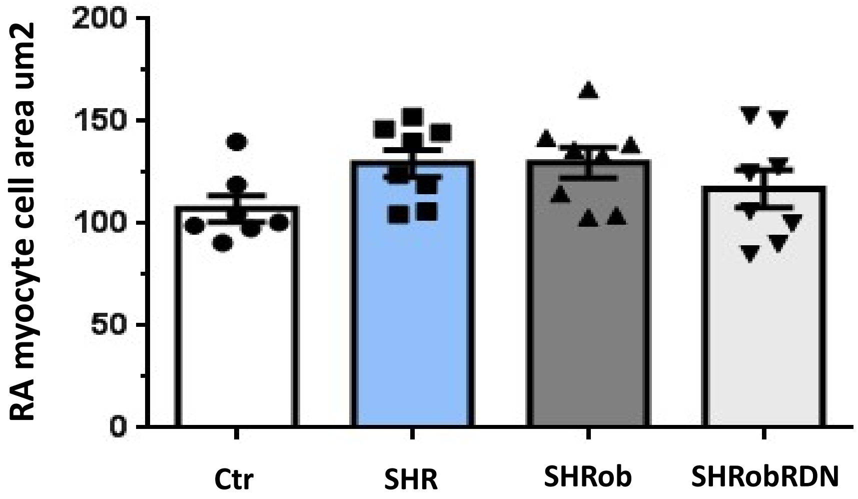

Supplementary Figure 2

C

Right atrial interstitial fibrosis

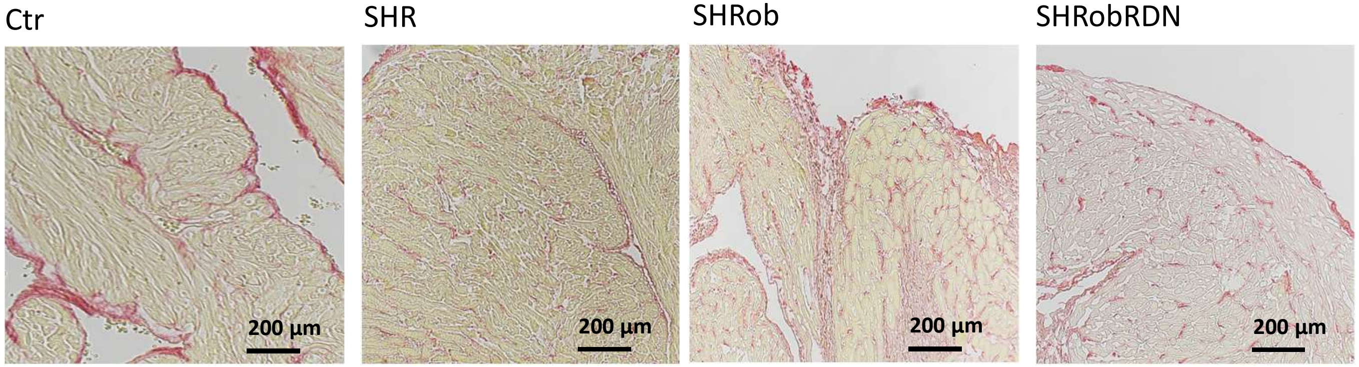

D

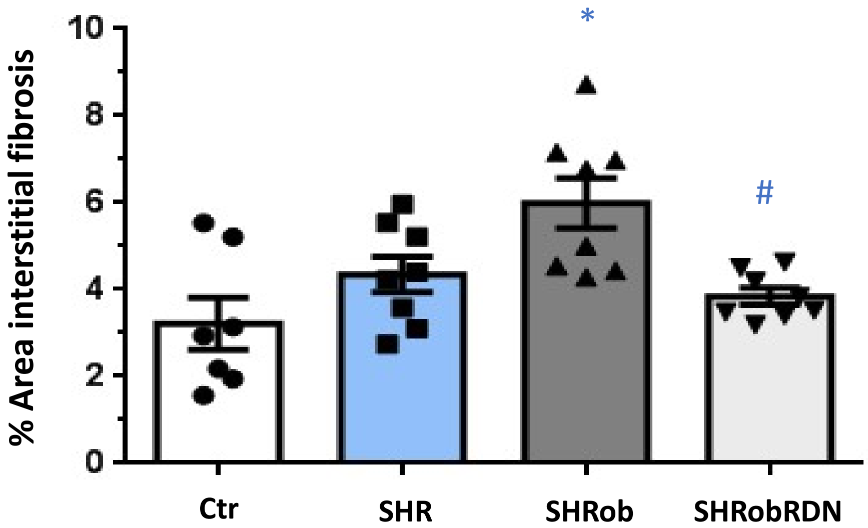

Supplementary
Figure 2

E

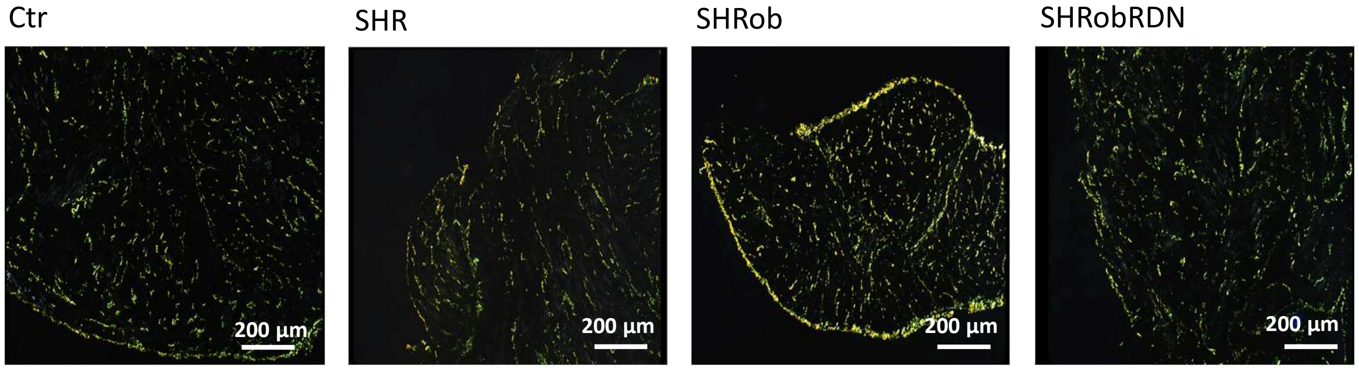

F

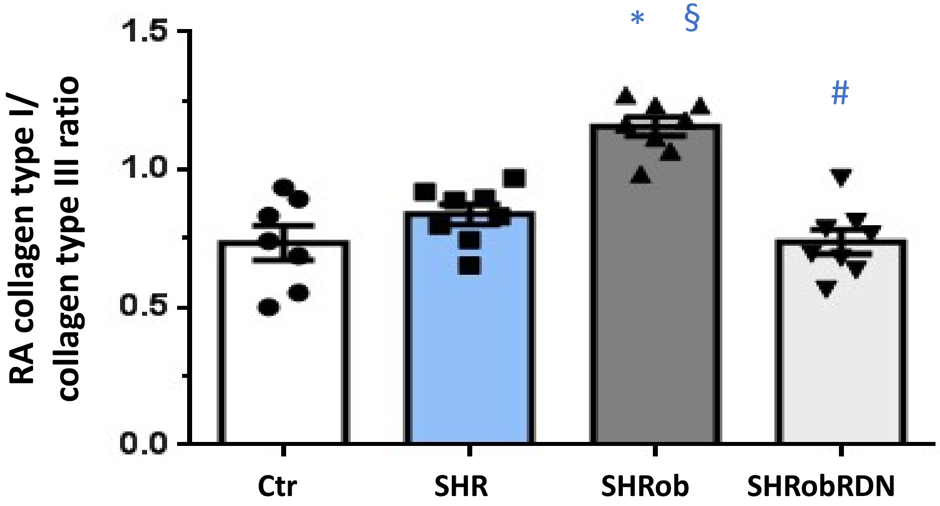

Supplementary Figure 2

G

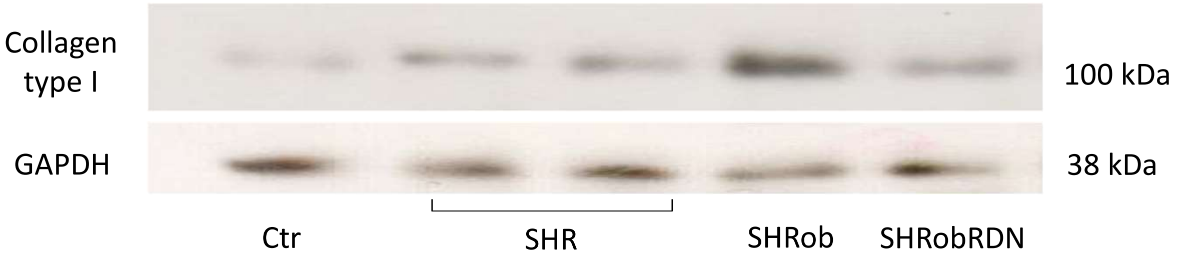

H

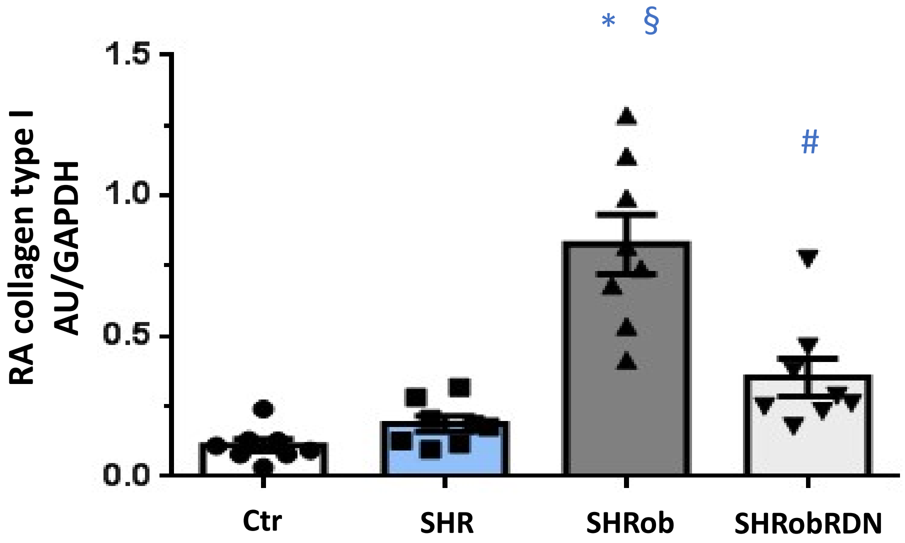

Supplementary Figure 3

A

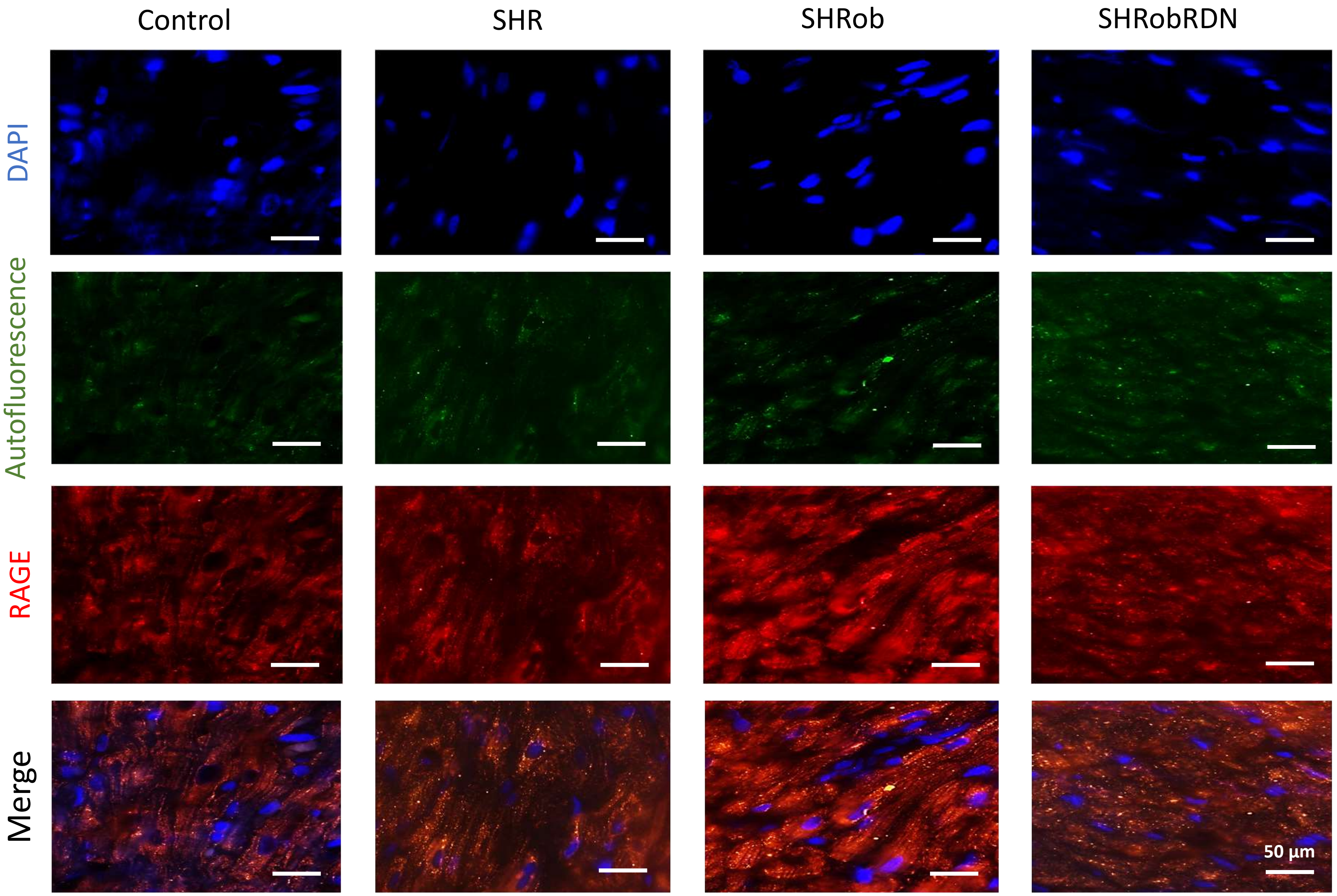

Supplementary Figure 3

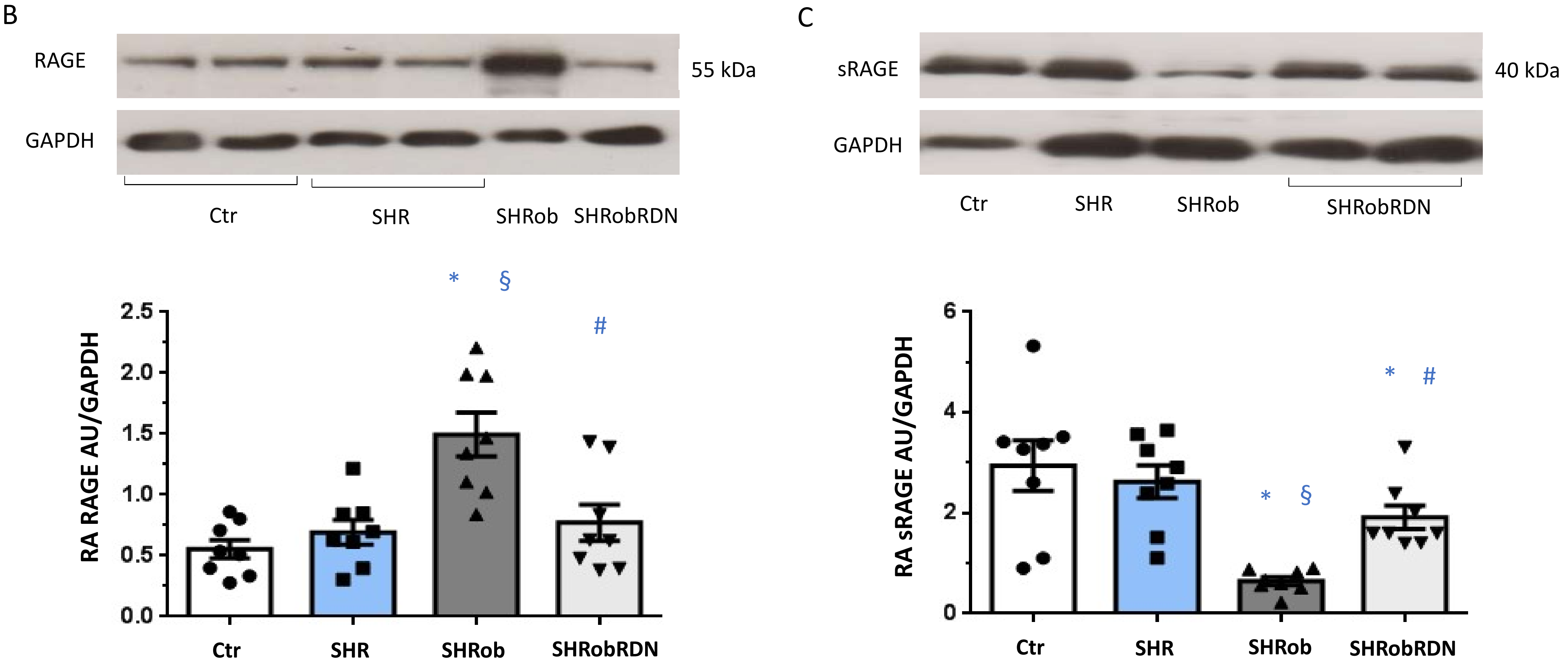

Supplementary
Figure 4

A Right atrial CML

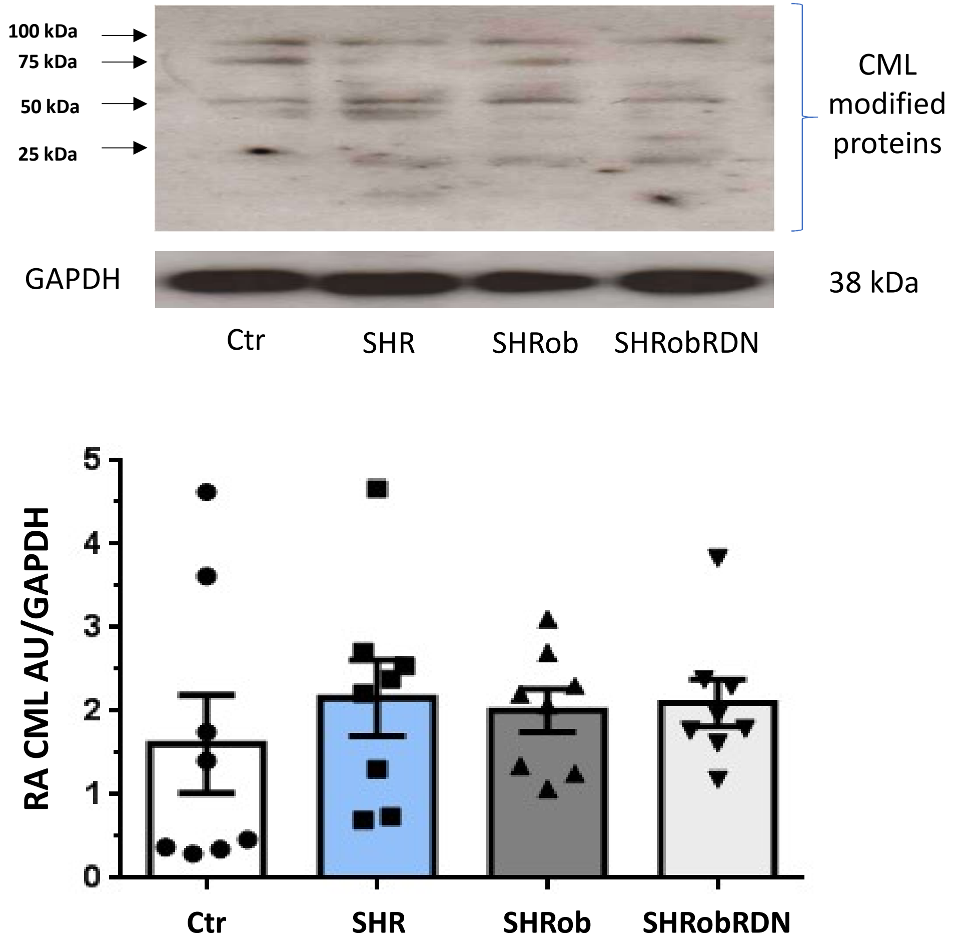

B Right atrial HMGB1

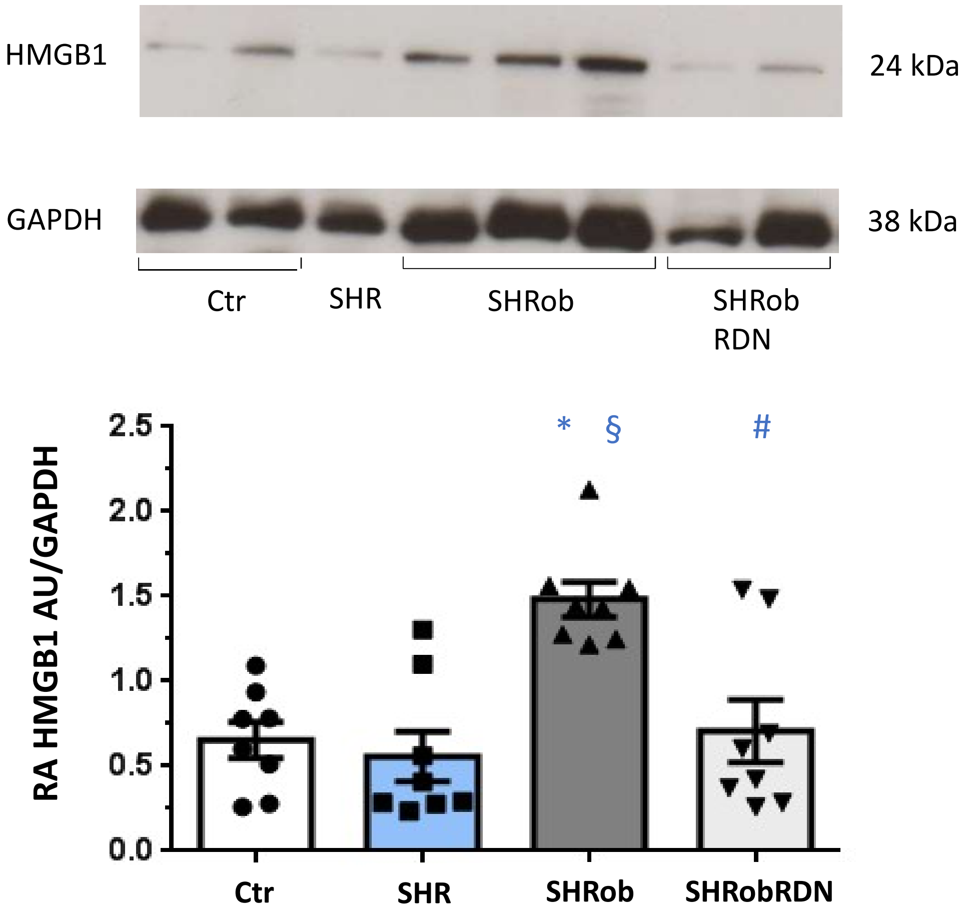

Supplementary
Figure 4

C Right atrial expression of NFkB

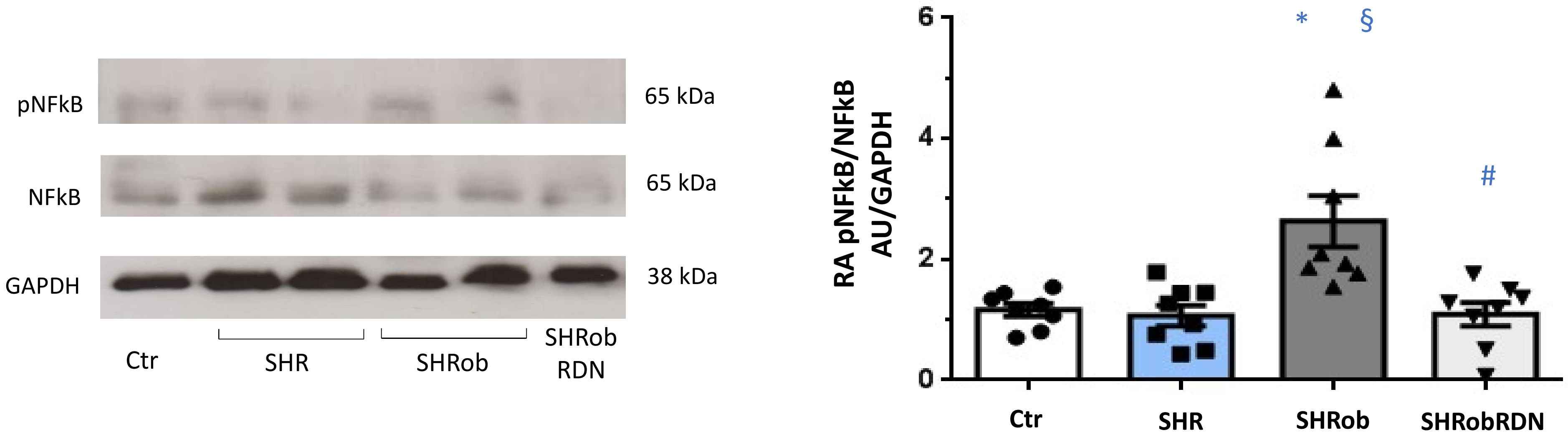

Supplementary
Figure 4

D Right atrial expression of IL-6

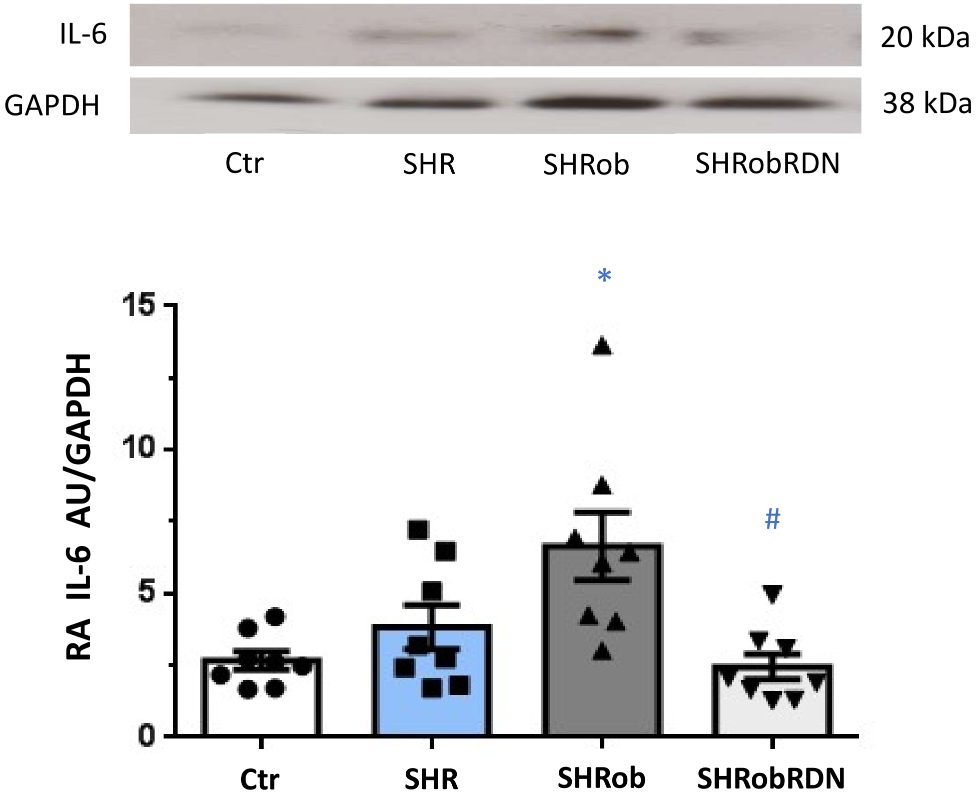

E Right atrial expression of TNFα

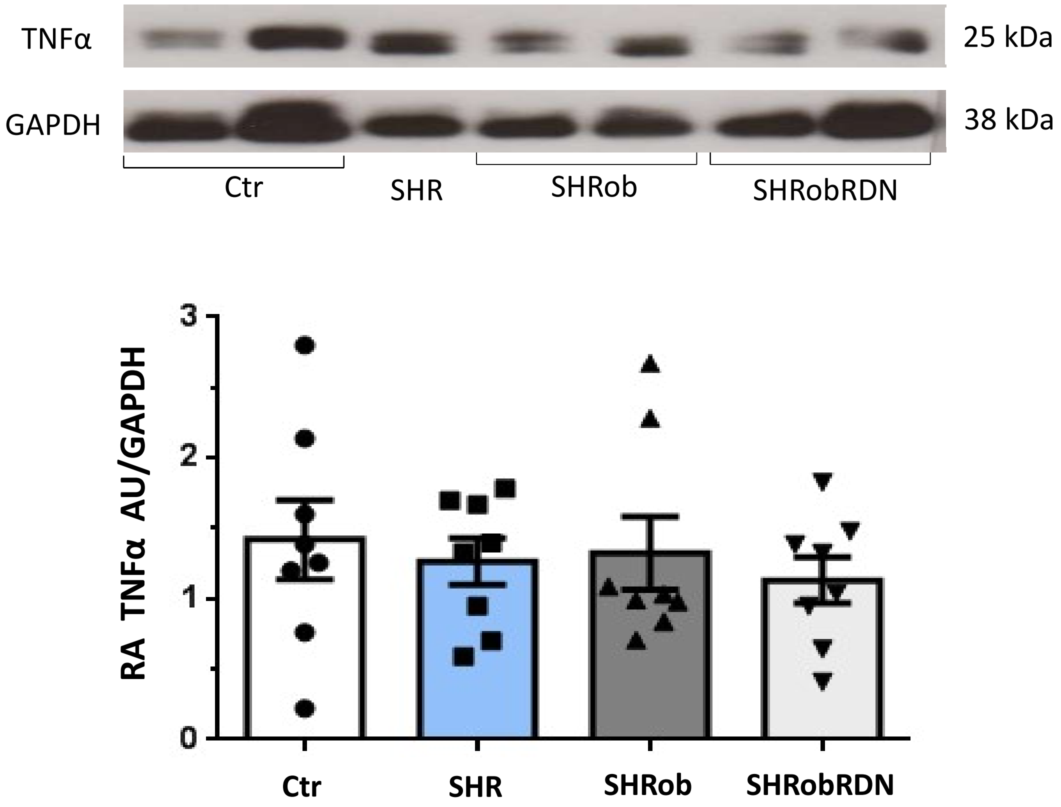

Supplementary
Figure 4

F Right atrial F4/80 + macrophages

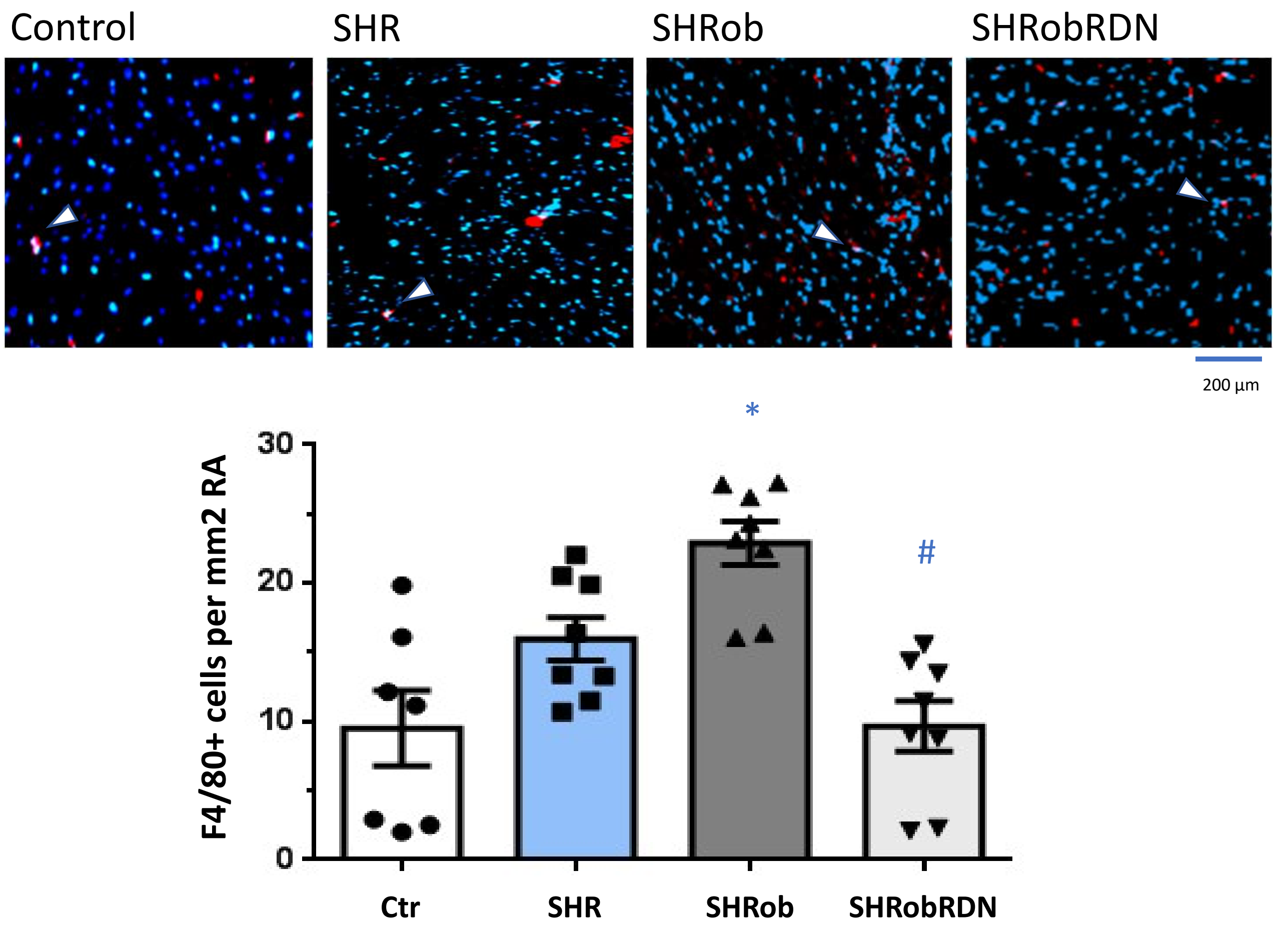

Supplementary
Figure 4

G Right atrial Ly6G+ cells

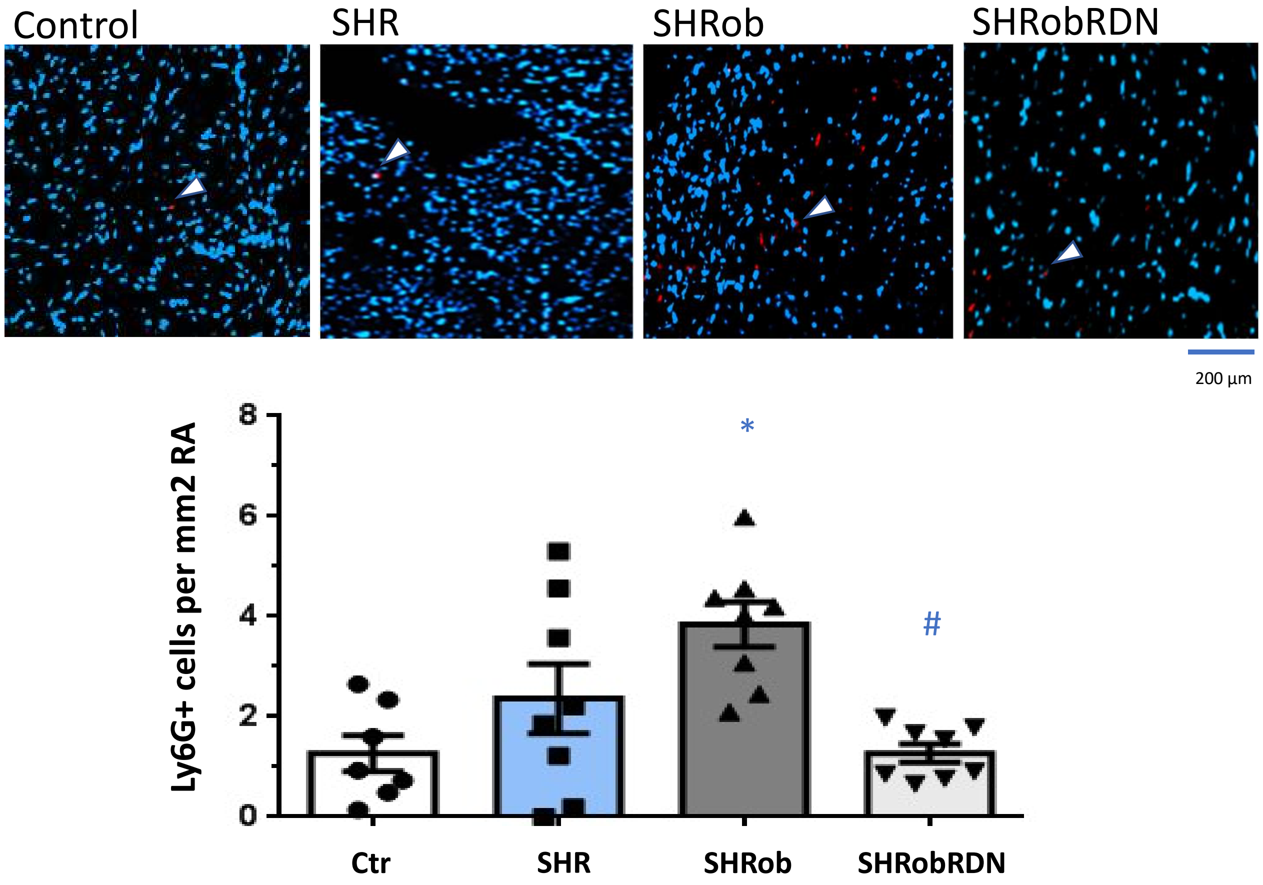

Supplement: Supplementary file 1 — Supplementary file1 (PDF 4351 KB) [file 395_2022_943_MOESM1_ESM.pdf]
