# Supplementary material for: Renal denervation reduces atrial remodeling in hypertensive rats with metabolic syndrome
Source: Basic Res Cardiol. 2022 Jul 14;117(1):36. doi: 10.1007/s00395-022-00943-6 (PMC9283368; doi:10.1007/s00395-022-00943-6)
Supplement: Supplementary file 2 — Supplementary file2 (DOCX 15 KB) [file 395_2022_943_MOESM2_ESM.docx]

**Supplementary Fig. 1:**

**a)** Representative images of immunofluorescence stainings (FITC green) of right atrial (RA) tyrosine hydroxylase (TH) positive nerve fibers (pointed to by white arrowheads in the enlarged image sections) and **b)** quantification of TH+ nerve fibers per cardiomyocyte in normotensive controls (n=7), SHR (n=8), SHRob (n=8) and SHRobRDN (n=8). **p<0.05 versus Control; #p<0.05 versus SHRob.*

**Supplementary Fig. 2:**

**a)** Representative histological pictures (hematoxyline eosin staining; scale bar 50 µm) and **b)** quantification of myocyte cell surface in right atrial (RA) tissue of normotensive controls (n=7), SHR (n=8), SHRob (n=8) and SHRobRDN (n=8). **c)** Representative histological pictures (Picro Sirius red staining; scale bar 200 µm) and **d)** quantification of right atrial fibrotic area (interstitial fibrillar collagen fractional area (%)) in normotensive controls Ctr (n=7), SHR (n=8), SHRob (n=8) and SHRobRDN (n=8). **e)** Representative images (polarization microscopy; scale bar 200 µm) and **f)** assessment of collagen type I (red-yellow birefringence)/collagen type III (green birefringence) ratio in right atrial tissue of normotensive controls (n=7), SHR (n=8), SHRob (n=8) and SHRobRDN (n=8). **g)** Representative Western blot and **h)** quantification of collagen type I in right atrial homogenates from normotensive controls (n=8), SHR (n=8), SHRob (n=8) and SHRobRDN (n=8). Collagen type I in arbitrary units (AU) normalized to GAPDH. **p<0.05 versus Control; § p<0.05 versus SHR; #p<0.05 versus SHRob.*

**Supplementary Fig. 3:**

**a)** Representative fluorescent immunostaining images of RAGE (TRITC red) in the right atria (RA) of controls (n=7), SHR (n=8), SHRob (n=8) and SHRobRDN (n=8). Scale bar=50 µm. Single channel breakdown with merged images. Representative Western blots (**b)** and **c)** upper panels) and quantification of **b)** right atrial RAGE (lower left panel) and **c)** sRAGE (lower right panel) in right atrial homogenates from normotensive controls Ctr (n=8), SHR (n=8), SHRob (n=8) and SHRobRDN (n=8). RAGE and sRAGE in arbitrary units (AU) normalized to GAPDH. **p<0.05 versus Control; § p<0.05 versus SHR; #p<0.05 versus SHRob.*

**Supplementary Fig. 4:**

**a)** Representative Western blot of right atrial (RA) CML-modified proteins (upper panel) and quantification of right atrial CML (lower panel) in normotensive controls (n=8), SHR (n=8), SHRob (n=8) and SHRobRDN (n=8). CML in arbitrary units (AU) normalized to GAPDH. The antibody specifically recognizes carboxymethyllysine modified proteins and reacts species independently (Western blot with multiple bands belonging to different proteins). **b)** Representative Western blot (upper panel) and quantification of right atrial HMGB1 (lower panel) in homogenates from normotensive controls (n=8), SHR (n=8), SHRob (n=8) and SHRobRDN (n=8). HMGB1 in arbitrary units (AU) normalized to GAPDH. **c)** Representative Western blots (left panel) and assessment of phospho-NFkB/NFkB ratio (right panel) in right atrial homogenates from normotensive controls (n=8), SHR (n=8), SHRob (n=8) and SHRobRDN (n=8). **d)** Representative Western blot (upper panel) and quantification of right atrial IL-6 (lower panel) in homogenates from normotensive controls (n=8), SHR (n=8), SHRob (n=8) and SHRobRDN (n=8). IL-6 in arbitrary units (AU) normalized to GAPDH. **e)** Representative Western blot (upper panel) and quantification of right atrial TNFα (lower panel) in homogenates from normotensive controls (n=8), SHR (n=8), SHRob (n=8) and SHRobRDN (n=8). TNFα in arbitrary units (AU) normalized to GAPDH.

Representative images of immunofluorescence stainings (TRITC red) for **f)** F4/80 macrophages (upper panel; example target cells pointed to by white arrowheads; scale bar 200 µm) and quantification of F4/80+ cells per mm2 RA area (lower panel). Nuclei of cells were stained with DAPI (blue). Representative images of immunofluorescence stainings (TRITC red) for **g)** Ly6G+ neutrophils (upper panel; example target cells pointed to by white arrowheads; scale bar 200 µm) and quantification of Ly6G+ cells per mm2 RA area (lower panel) in normotensive controls (n=7), SHR (n=8), SHRob (n=8) and SHRobRDN (n=8) rats. Nuclei of cells were stained with DAPI (blue). **p<0.05 versus Control; § p<0.05 versus SHR; #p<0.05 versus SHRob.*
