# Supplementary material for: Renal denervation reduces atrial remodeling in hypertensive rats with metabolic syndrome
Source: Basic Res Cardiol. 2022 Jul 14;117(1):36. doi: 10.1007/s00395-022-00943-6 (PMC9283368; doi:10.1007/s00395-022-00943-6)
Supplement: Supplementary file 3 — Supplementary file3 (DOCX 28 KB) [file 395_2022_943_MOESM3_ESM.docx]

|  | **Ctr** | **SHR** | **SHRob** | **SHRobRDN** | **Ctr**  **vs.**  **SHR** | **Ctr**  **vs.**  **SHRob** | **Ctr**  **vs. SHRobRDN** | **SHR**  **vs.**  **SHRob** | **SHR**  **vs.**  **SHRobRDN** | **SHRob**  **vs.**  **SHRobRDN** |
| --- | --- | --- | --- | --- | --- | --- | --- | --- | --- | --- |
|  |  |  |  |  | p-values | | | | | |
| RA RAGE [AU/GAPDH] | 0.55±0.1 (n=8) | 0.68±0.13 (n=8) | 1.34±0.18 (n=8) | 0.77±0.15 (n=8) | 0.95 | **0.008** | 0.78 | **0.03** | 0.98 | **0.045** |
| RA sRAGE [AU/GAPDH] | 3.33±0.55 (n=8) | 2.45±0.31 (n=8) | 0.65±0.08 (n=8) | 1.7±0.17 (n=8) | 0.18 | **<0.0001** | **0.0026** | **0.0004** | 0.18 | **0.048** |
| RA CML [AU/GAPDH] | 1.59±0.8 (n=8) | 2.29±0.6 (n=8) | 1.95±0.3 (n=8) | 2.03±0.4 (n=8) | 0.81 | 0.97 | 0.95 | 0.98 | 0.99 | 1.0 |
| RA HMGB1 [AU/GAPDH] | 0.61±0.1 (n=8) | 0.56±0.15 (n=8) | 1.44±0.1 (n=8) | 0.78±0.23 (n=8) | 0.99 | **0.0014** | 0.85 | **0.0005** | 0.71 | **0.019** |
| RA Collagen type I [AU/GAPDH] | 0.08±0.02 (n=8) | 0.18±0.02 (n=8) | 0.87±0.14 (n=8) | 0.4±0.08 (n=8) | 0.87 | **0.0001** | 0.13 | **0.0001** | 0.32 | **0.0065** |
| RA Collagen type I/collagen type III ratio | 0.74±0.1 (n=7) | 0.88±0.04 (n=8) | 1.17±0.03 (n=8) | 0.78±0.04 (n=8) | 0.25 | **<0.0001** | 0.92 | **0.0032** | 0.47 | **<0.0001** |
| RA phosphoNFkB/  total NFkB ratio | 1.16±0.09 (n=8) | 1.22±0.19 (n=8) | 2.51±0.39 (n=8) | 0.98±0.3 (n=8) | 1.0 | **0.0084** | 0.98 | **0.02** | 0.96 | **0.0091** |
| RA IL6  [AU/GAPDH] | 2.65±0.3 (n=8) | 3.83±0.8 (n=8) | 6.64±1.2 (n=8) | 2.61±0.48 (n=8) | 0.76 | **0.014** | 1.0 | 0.08 | 0.72 | **0.009** |
| RA TNFa  [AU/GAPDH] | 1.6±0.6 (n=8) | 1.25±0.2 (n=8) | 1.25±0.2 (n=8) | 1.16±0.2 (n=8) | 0.84 | 0.83 | 0.75 | 1.0 | 1.0 | 1.0 |
| RA F4/80+  macrophages per mm2 | 10.4±3.6 (n=7) | 15.1±1.9 (n=8) | 22.3±1.7 (n=8) | 7.9±2.5 (n=8) | 0.28 | **0.0011** | 0.99 | 0.09 | 0.19 | **0.0009** |
| RA Ly6G+  neutrophils per mm2 | 0.9±0.3 (n=7) | 1.7±0.8 (n=8) | 3.7±0.6 (n=8) | 1.3±0.2 (n=8) | 0.75 | **0.026** | 0.97 | 0.09 | 0.93 | **0.036** |
| RA Interstitial Fibrosis [%] | 3.2±0.6 (n=7) | 4.4±0.6 (n=8) | 5.8±0.6 (n=8) | 3.85±0.2 (n=8) | 0.48 | **0.0069** | 0.8 | 0.36 | 0.89 | **0.041** |
| RA atrial myocyte cell area [um2] | 109 ± 7 (n=7) | 120 ± 8 (n=8) | 117 ± 8 (n=8) | 117 ± 10 (n=8) | 0.74 | 0.91 | 0.91 | 0.99 | 0.99 | 1.0 |
| RA TH+ nerve fibers per cardiomyocyte | 0.32±0.02 (n=7) | 0.38±0.03 (n=8) | 0.44±0.03 (n=8) | 0.29±0.04 (n=8) | 0.54 | **0.034** | 0.86 | 0.38 | 0.17 | **0.0051** |

**Supplementary Table 1: Right atrial interstitial remodeling, RAGE/sRAGE, RAGE-ligands**

SHR: Spontaneously Hypertensive Rat; SHRob: Spontaneously Hypertensive Obese Rat; SHRobRDN: Spontaneously Hypertensive Obese Rat with renal denervation; AU: Arbitrary Units; RAGE: Receptor for Advanced Glycation End products; sRAGE: soluble Receptor for Advanced Glycation End products; CML: Carboxy-Methyl-Lysine; HMGB1: High Mobility Group Box1 protein; GAPDH: Glyceraldehyde 3-Phosphate Dehydrogenase; RDN: Renal Denervation; bpm: beats per minute; RA: right atrial; TH: tyrosine hydroxylase; IL-6: interleukin 6; TNFα: tumor necrosis factor α; NFkB: nuclear factor kappa-light-chain-enhancer of activated B-cells

Data are reported as mean +SEM. Bold values indicate statistically significant (p<0.05).
